# Blockade of microglial adenosine A_2A_ receptor suppresses elevated pressure‐induced inflammation, oxidative stress, and cell death in retinal cells

**DOI:** 10.1002/glia.23579

**Published:** 2019-01-22

**Authors:** Inês Dinis Aires, Raquel Boia, Ana Catarina Rodrigues‐Neves, Maria Helena Madeira, Carla Marques, António Francisco Ambrósio, Ana Raquel Santiago

**Affiliations:** ^1^ Coimbra Institute for Clinical and Biomedical Research (iCBR), Faculty of Medicine University of Coimbra Coimbra Portugal; ^2^ CNC.IBILI Consortium University of Coimbra Coimbra Portugal; ^3^ Association for Innovation and Biomedical Research on Light and Image (AIBILI) Coimbra Portugal

**Keywords:** adenosine A_2A_ receptors, glaucoma, microglia, neurodegeneration, neuroinflammation

## Abstract

Glaucoma is a retinal degenerative disease characterized by the loss of retinal ganglion cells and damage of the optic nerve. Recently, we demonstrated that antagonists of adenosine A_2A_ receptor (A_2A_R) control retinal inflammation and afford protection to rat retinal cells in glaucoma models. However, the precise contribution of microglia to retinal injury was not addressed, as well as the effect of A_2A_R blockade directly in microglia. Here we show that blocking microglial A_2A_R prevents microglial cell response to elevated pressure and it is sufficient to protect retinal cells from elevated pressure‐induced death. The A_2A_R antagonist SCH 58261 or the knockdown of A_2A_R expression with siRNA in microglial cells prevented the increase in microglia response to elevated hydrostatic pressure. Furthermore, in retinal neural cell cultures, the A_2A_R antagonist decreased microglia proliferation, as well as the expression and release of pro‐inflammatory mediators. Microglia ablation prevented neural cell death triggered by elevated pressure. The A_2A_R blockade recapitulated the effects of microglia depletion, suggesting that blocking A_2A_R in microglia is able to control neurodegeneration in glaucoma‐like conditions. Importantly, in human organotypic retinal cultures, A_2A_R blockade prevented the increase in reactive oxygen species and the morphological alterations in microglia triggered by elevated pressure. These findings place microglia as the main contributors for retinal cell death during elevated pressure and identify microglial A_2A_R as a therapeutic target to control retinal neuroinflammation and prevent neural apoptosis elicited by elevated pressure.

## 1 INTRODUCTION

Glaucoma, the second leading cause of irreversible blindness worldwide, is characterized by the loss of retinal ganglion cells (RGCs) and optic nerve atrophy (Vohra, Tsai, & Kolko, 2013). Microglia are the immune competent cells of the central nervous system. In resting state, microglia secrete supporting factors and present long protrusions that continuously scan retinal parenchyma (Karlstetter et al., 2015; Karlstetter, Ebert, & Langmann, 2010; Santos et al., 2008). Progressive neurodegeneration in glaucoma is accompanied by phenotypic alterations in microglial cells of the retina (Tezel, 2011).

The onset of the inflammatory response in glaucoma is hypothesized to be triggered by an altered crosstalk between RGCs and microglial cells, thus increasing the release of pro‐inflammatory mediators, namely tumor necrosis factor (TNF), interleukin‐1β (IL‐1β), reactive oxygen species (ROS), and nitric oxide (NO) (Almasieh, Wilson, Morquette, Cueva Vargas, & Di Polo, 2012; Chua et al., 2012; Lee, 2013; Madeira, Boia, Santos, Ambrósio, & Santiago, 2015). Therefore, strategies that modulate microglial cell response to damage have been suggested to afford neuroprotection in glaucoma (Almasieh et al., 2012; Bosco et al., 2008; Langmann, 2007; Madeira et al., 2015; Madeira, Boia, et al., 2015).

Adenosine is a neuromodulator in the central nervous system acting through the activation of four receptors, A_1_, A_2A_, A_2B_, and A_3_. We demonstrated that the blockade of adenosine A_2A_ receptor (A_2A_R) confers neuroprotection to the retina in glaucoma models through the control of microglia responsiveness (Boia et al., 2017; Boia, Ambrósio, & Santiago, 2016; Madeira et al., 2016; Madeira, Elvas, et al., 2015; Santiago et al., 2014). However, the specific role of microglial cells was not addressed, as well as the impact of A_2A_R blockade in microglial cells in the context of glaucoma. Since elevated intraocular pressure is considered the main risk factor of glaucoma, we focused on the contribution of microglia to the death of retinal neurons under elevated pressure to model glaucoma in vitro (Aires, Ambrósio, & Santiago, 2016). In addition, we also studied whether the pharmacological blockade or the genetic inactivation of A_2A_R in microglia could attenuate the inflammatory profile of microglia, using a murine microglia cell line, primary retinal microglia cultures, rat retinal neural cell cultures, and human retinal tissue exposed to elevated pressure.

## 2 MATERIALS AND METHODS

### 2.1 Animals

All experiments using animals were approved by the Animal Welfare Committee of the Faculty of Medicine of University of Coimbra and conducted in accordance with the European Community directive guidelines for the use of animals in laboratory (2010/63/EU) transposed to the Portuguese law in 2013 (Decreto‐Lei 113/2013), and in agreement with the Association for Research in Vision and Ophthalmology statement for animal use.

### 2.2 Primary retinal neural cell cultures

Primary retinal neural cell cultures were prepared from 3 to 5 days old Wistar rats, as described previously (Santiago, Cristovao, Santos, Carvalho, & Ambrósio, 2007). The cells were plated at a density of 2 × 10^6^ cells/cm^2^ in 12‐well plates with glass coverslips, or 1.7 × 10^6^ cells/cm^2^ in 6‐well plates, all precoated with poly‐d‐lysine (0.1 mg/mL). Cells were cultured at 37°C in a humidified atmosphere of 5% CO_2_ for 7 days. The primary retinal neural cell culture is composed of retinal neurons, Müller cells, astrocytes, and microglia.

### 2.3 Primary retinal microglial cell cultures

Primary retinal microglial cell cultures were obtained from 3 to 4 days old Wistar rats, as described previously (Li, Qu, & Wang, 2015), with some modifications, as follows. The retinas were dissociated, and the cell suspension was plated in uncoated T75 flasks (corresponding to four retinas in each flask) and maintained in Dulbecco's Modified Eagle Medium/Nutrient Mixture F‐12 (DMEM/F‐12) with GlutaMAX™ (GIBCO, Invitrogen, Carlsbad, CA) supplemented with 10% FBS (GIBCO, Invitrogen, Carlsbad, CA), 100 U/mL penicillin, and 100 μg/mL streptomycin (GIBCO, Invitrogen, Carlsbad, CA). The culture medium was replaced every week. After 2 weeks, the cultures were shaken for 1 hr at 110 g and microglial cells were collected from the supernatant. Culture medium was added to the remaining adherent cells, and microglia were collected every 1–2 weeks, in a total of four collections.

Microglia were plated at a density of 5.3 × 10^4^ cells/well in 24‐well plates with glass coverslips coated with poly‐d‐lysine (0.1 mg/mL) and cultured at 37°C in a humidified atmosphere of 5% CO_2_. The purity of the culture (96%) was assessed by immunocytochemistry with anti‐CD11b antibody.

### 2.4 BV‐2 cell line

BV‐2 cells (murine microglia; ICLC Cat# ATL03001, RRID:CVCL_0182) were maintained in Roswell Park Memorial Institute (RPMI) medium (GIBCO, Invitrogen, Carlsbad, CA) supplemented with 10% FBS (GIBCO, Invitrogen, Carlsbad, CA) and antibiotics (100 U/mL penicillin, 100 μg/mL streptomycin; GIBCO, Invitrogen, Carlsbad, CA). For experiments, BV‐2 cells were cultured in RPMI supplemented with 2% FBS and antibiotics (100 U/mL penicillin, 100 μg/mL streptomycin) at a density of 6 × 10^3^ cells/cm^2^ in six‐well plates or 1 × 10^5^ cells/cm^2^ in 12‐well plates, and cultured at 37°C in a humidified atmosphere of 5% CO_2_.

### 2.5 Human organotypic retinal cultures

Human retinas were obtained from postmortem donors (age range: 42–78 years old) from the Coimbra Hospital and University Centre, in accordance with the Declaration of Helsinki for research involving human tissue. The experiments were approved by the Ethical Committee of the Faculty of Medicine, University of Coimbra. Human retinas (until 12 hr of postmortem time) were dissected in Ca^2+^‐ and Mg^2+^‐free Hank's balanced salt solution (HBSS, in mM: 137 NaCl, 5.4 KCl, 0.45 KH_2_PO_4_, 0.34 Na_2_HPO_4_, 4 NaHCO_3_, 5 glucose; pH 7.4). Retinal pieces with similar sizes were cultured in transwell inserts with a 0.4 μm pore diameter (Millipore Bioscience Research Reagents, Billerica, MA) with the ganglion cell layer facing up in Neurobasal‐A medium (GIBCO, Invitrogen, Carlsbad, CA) supplemented with 2% FBS, 2% B27, 200 mM l‐glutamine and antibiotics (Antibiotic–Antimycotic Solution, Sigma‐Aldrich, St Louis, MO), as described previously (Niyadurupola, Sidaway, Osborne, Broadway, & Sanderson, 2011; Portugal et al., 2017). Immediately after dissection, retinal pieces were placed in a standard cell incubator with 5% CO_2_ for 1 hr before beginning the experiment. From each donor, the three experimental conditions were prepared in duplicate, thus allowing an internal control per experiment.

### 2.6 Cultures treatment

Cell cultures were incubated with 50 nM of the selective A_2A_R antagonist SCH 58261 (Tocris Bioscience, Bristol, UK) 45 min before placing the cultures inside the pressure chamber.

In BV‐2 microglial cells, the knockdown of A_2A_R was accomplished by small interfering ribonucleic acid (siRNA). Cells were transfected with 12 pmol of siRNA against Adora2A (siRNA ID: s62046) or with nontargeting control sequences (Silencer® Select negative control #1 siRNA; Ambion, Foster City, CA). Briefly, cells were plated in Opti‐MEM® (GIBCO, Invitrogen, Carlsbad, CA) at a density of 6 × 10^3^ cells/cm^2^ and then incubated with siRNA for 48 hr (medium replacement and reinforcement of siRNA at 24 hr). The transfections were performed using lipofectamine® RNAiMAX reagent (Invitrogen, Carlsbad, CA) according to the manufacturer's instructions.

The impact of the pro‐inflammatory cytokines TNF and IL‐1β on retinal cell death induced by EHP was assessed by preincubating cells (45 min prior exposure to EHP) with rabbit anti‐TNF (2 μg/mL; PeproTech, London, UK) and goat anti‐IL‐1β (1 μg/mL; R&D Systems, Minneapolis, MN) antibodies.

Microglial contribution to EHP‐induced cell death was elucidated by depleting microglia from primary retinal neural cell cultures before exposure to EHP. Briefly, on the third day in culture, primary retinal neural cell cultures were incubated with 2% clodronate‐loaded liposomes (Van Rooijen & Sanders, 1994) in fresh culture medium for 24 hr. Then, the cell culture medium was replaced by previously collected supernatant.

Cultures were submitted to elevated hydrostatic pressure (EHP; 70 mmHg above atmospheric pressure) for 4 hr or 24 hr, as previously described (Madeira, Elvas, et al., 2015; Sappington, Chan, & Calkins, 2006). Control cultures were kept at atmospheric pressure in a standard cell incubator.

### 2.7 Western blot

Protein extracts were prepared in ice‐cold radioimmunoprecipitation assay (RIPA) buffer with 1 mM of dithiothreitol (Sigma‐Aldrich, St. Louis, MO) and complete mini protease inhibitor cocktail tablets (Roche, Sigma‐Aldrich, St. Louis, MO). Western blot was performed as previously described (Baptista et al., 2014). Membranes were probed with the antibodies indicated in Table 1. Blots were developed with ECL (Clarity™ from Bio‐Rad, CA) and WesternBright Sirius from Advansta, Menlo Park, CA) or with ECF™ (GE Healthcare Amersham™, Little Chalfont, UK), in accordance with the manufacturer's instructions. Membranes were reprobed for glyceraldehyde‐3‐phosphate dehydrogenase (GAPDH) as a loading control.

**Table 1:** List of primary and secondary antibodies used in this work

	Supplier	Host	Dilution	Technique
Primary antibodies				
Anti‐A_2A_R	Santa Cruz biotechnology Cat# sc‐32,261 RRID:AB_2226517	Rabbit	1:200	Western blot
Anti‐GAPDH	SICGEN Cat# AB0049‐200 RRID:AB_2333141	Goat	1:5,000	Western blot
Anti‐A_2A_R	Santa Cruz biotechnology Cat# sc‐7,504 RRID:AB_2273960	Goat	1:50	Microscopy
Anti‐CD11b	Bio‐Rad/AbD Serotec Cat# MCA275GA RRID:AB_566455	Mouse	1:100	Microscopy
Anti‐Iba‐1	Wako Cat# 019‐19741 RRID:AB_839504	Rabbit	1:500	Microscopy
Anti‐TNF	Peprotech Cat# 500‐P72 RRID:AB_147663	Rabbit	2 μg/mL	Cell treatment
Anti‐IL‐1β	R&D Systems Cat# AF‐501‐NA RRID:AB_354508	Goat	1 μg/mL	Cell treatment
Secondary antibodies				
Anti‐rabbit IgG (H + L) secondary antibody, HRP	Bio‐Rad / AbD Serotec Cat# 1706515 RRID:AB_2617112	Goat	1:10,000	Western blot
Anti‐goat IgG (H + L) secondary antibody, AP	Thermo Fisher Scientific Cat# 61–1,620 RRID:AB_2533922	Rabbit	1:10,000	Western blot
Alexa Fluor anti‐mouse 568	Thermo Fisher Scientific Cat# A10037 RRID:AB_2534013	Donkey	1:200	Microscopy
Alexa Fluor anti‐mouse 488	Thermo Fisher Scientific Cat# A11001 RRID:AB_2534069	Goat	1:200	Microscopy
Alexa Fluor anti‐rabbit 568	Thermo Fisher Scientific Cat# A11036 RRID:AB_10563566	Goat	1:200	Microscopy
Alexa Fluor anti‐goat 488	Thermo Fisher Scientific Cat# A‐11078 RRID:AB_2534122	Rabbit	1:200	Microscopy

### 2.8 Immunolabeling

Cell cultures were immunostained as previously described (Madeira, Boia, et al., 2016), using the antibodies listed in Table 1. Human organotypic retinal cultures, following fixation with 4% PFA with 4% sucrose solution for 10 min, were blocked with 1% Triton X‐100, 1% BSA and 10% goat serum, in PBS, for 60 min. Primary antibodies (Table 1) were incubated overnight at 4°C. Retinal explants were washed with PBS and incubated with the secondary antibodies (Table 1) overnight at 4°C. Cultures were washed with PBS and incubated with 4′,6‐diamidine‐2′‐phenylindole dihydrochloride (DAPI; Invitrogen, Carlsbad, CA; 1:2000) for 10 min. The preparations were washed with PBS and mounted with Glycergel mounting medium (DAKO, Agilent, Santa Clara, CA). In all experiments, cells were observed with a confocal microscope (LSM 710, Zeiss, Oberkochen, DE), and from each condition, five random fields were acquired (20× objective), unless otherwise mentioned.

### 2.9 Densitometric analysis of A_2A_R immunoreactivity

Quantitative analysis of total fluorescence was performed in the images of primary retinal cell cultures immunostained for A_2A_R and cluster of differentiation molecule 11b (CD11b). A_2A_R immunoreactivity was analyzed with the public domain ImageJ program (http://rsb.info.nih.gov/ij/; RRID:SCR_003070). The corrected total cell fluorescence (CTCF), was calculated with the formula, as previously described (Madeira, Elvas, et al., 2015):

CTCF = Integrated density − (Area of selected cell × Mean fluorescence background reading).

### 2.10 Morphometric analysis of microglia

Morphological alterations in microglia from primary retinal neural cultures were assessed as previously described (Kurpius, Wilson, Fuller, Hoffman, & Dailey, 2006). First, threshold was arbitrarily but uniformly applied to confocal images labeled with CD11b. Next, the particle measurement feature of ImageJ (http://rsb.info.nih.gov/ij/) was used to automatically measure the circularity index. A circularity value of 1.0 indicates perfect circular cell and values near to zero indicate elongated cells.

In human organotypic retinal cultures, microglia morphology was determined from 3D reconstruction images acquired in a confocal microscope (LSM 710, Zeiss, Oberkochen, DE) using a 40× objective and Z‐stack interval of 1 μm. Iba‐1 immunoreactive cells were evaluated from four human donors, in a total of 21–28 cells analyzed per condition. Microglia reconstruction was performed using Simple Neurite Tracer open source software from Fiji‐ImageJ, available at (http://fiji.sc/Simple_Neurite_Tracer:_Step-By-Step_Instructions; RRID:SCR_002074), as previously described (Tavares et al., 2017). From this analysis, the morphologic parameters assessed were a total number of processes, total length, last intersection radius, and Sholl analysis.

### 2.11 Dihydroethidium staining

The production of reactive oxygen species (ROS) was assessed by dihydroethidium (DHE) (Invitrogen, Carlsbad, CA) staining (Reyes, Brennan, Shen, Baldwin, & Swanson, 2012). Culture medium was collected and the cells were incubated with 5 μM DHE prepared in fresh culture medium for 1 hr in the cell incubator. Then, the medium with DHE was replaced by the previous collected medium and the cultures were maintained for 4 hr in EHP or in control conditions. The cell cultures were rinsed with warm PBS and fixed.

### 2.12 Griess reaction assay

The production of NO was assessed by the Griess reaction method. The culture medium was collected and centrifuged to remove cell debris and then incubated (1:1) with the Griess reagent mix (1% sulfanilamide in 5% phosphoric acid with 0.1% N‐1‐naphtylenediamine) for 30 min, protected from light. The optical density was measured at 550 nm using a microplate reader (Synergy HT; Biotek, Winooski, VT). The nitrite concentration was determined by comparison to a sodium nitrite standard curve.

### 2.13 Cell proliferation assay

Cell proliferation was assessed using the Click‐iT® EdU cell proliferation assay according to the instructions provided by the manufacturer (Life Technologies, Carlsbad, CA). Cells were immunostaining for CD11b as previously described. Nuclei were stained with Hoechst 33342 (1:2000).

### 2.14 Terminal deoxynucleotidyl transferase‐mediated deoxyuridine triphosphate nick end labeling (TUNEL) assay

Apoptotic cells were detected using DeadEnd™ Fluorometric TUNEL System following the manufacturer's instructions (Promega, Madison, WI). Microglial cells were labeled with anti‐CD11b antibody and the nuclei were stained with DAPI (1:2,000).

### 2.15 Phagocytosis assays

Phagocytosis was assayed with fluorescent latex beads (1 μm diameter) in BV‐2 cells and primary microglia as we previously described (Madeira, Boia, et al., 2016), with minor modifications as follows. Cells were incubated with 0.025% beads for 60 min at 37°C. In the end, cells were fixed, and BV‐2 cells were stained with phalloidin conjugated to tetramethylrhodamine B isothiocyanate (Phalloidin‐TRITC, 1:500; Sigma‐Aldrich, St Louis, MO) or labeled with anti‐CD11b in primary microglia using the antibodies described in Table 1. Nuclei were stained with DAPI (1:2,000). The phagocytic efficiency (Phago Eff.) was calculated with the following formula, as previously described (Madeira, Boia, et al., 2016; Pan et al., 2011).

$$\text{Phago}\;\mathrm{Eff}.\left(\%\right)=\frac{\left(1\times x1+2\times x2+3\times x3\ldots +n\times \mathrm{xn}\right)}{\text{Total number of cells}}\times 100\%$$

*xn* represents the number of cells containing *n* beads (*n* = 1,2,3, … up to a maximum of 6 points for more than 5 beads per cell).

In primary retinal microglia, phagocytosis was also evaluated with dead cells. Primary retinal neural cell cultures were exposed to UV light (200–280 nm) for 30 min and then cultured overnight. Dying/dead cells were then labeled with 1 μg/mL of propidium iodide (PI) and washed twice with PBS. The number of PI^+^ cells was counted and 5 × 10^4^ cells/mL were added to microglia 1 hr before the end of the experiments. Microglial cells were washed, fixed, and then immunolabeled using the CD11b antibody (Table 1). Nuclei were stained with DAPI (1:2,000).

### 2.16 Scratch wound assay

Confluent BV‐2 cells, plated in six‐well plates, were wounded with a sterile p200 pipet tip and washed to remove nonadherent cells. Cells were subsequently cultured for 4 hr in control or EHP conditions. Images (before, immediately after and 4 hr after the wound) were acquired with an inverted fluorescence microscope (Zeiss Axio HXP‐120, Zeiss, Oberkochen, DE). The number of cells in the scratch before was subtracted to the number of cells in the scratch after exposure to EHP for 4 hr in five random fields acquired in each condition.

### 2.17 Boyden chamber migration assay

Microglia migration was assessed in BV‐2 cells and in primary microglia using the Boyden chamber migration assay as described (Siddiqui, Lively, Vincent, & Schlichter, 2012). BV‐2 cells were kept in serum‐free medium for 24 hr before beginning the experiment. Cells (BV‐2 cells in 2% FBS or primary microglia in 10% FBS) were plated in transwell culture inserts with 8.0 μm pore diameter (Merck, Millipore, Billerica, MA) at a density of 3 × 10^4^ cells/cm^2^ and then cultured for 4 hr in control or EHP conditions. Inserts were washed with warm PBS and following fixation in 4% paraformaldehyde with 4% sucrose for 10 min the cells in upper side were removed with a cotton swab. Nuclei were stained with DAPI (1:2,000) to allow cell counting. The membrane was removed and mounted with Glycergel in glass slides. The preparations were observed in an inverted fluorescence microscope (Zeiss Axio HXP‐120, Oberkochen, DE) and five random images per preparation were acquired (10× objective). The number of cells in the bottom side of the insert (the cells that migrated) was counted.

### 2.18 Real‐time quantitative polymerase chain reaction

The cells were washed with ice‐cold RNAse‐free PBS and total RNA was extracted using the TRIZOL reagent (Life Technologies, Carlsbad, CA). RNA concentration and purity were determined using NanoDrop®. Resulting RNA samples were treated with Deoxyribonuclease I (DNase I—amplification grade; Life Technologies, Carlsbad, CA) to eliminate possible contamination with genomic DNA. RNA was reversed transcribed to cDNA using NZY M‐MuLV First‐Strand cDNA Synthesis Kit, according to supplier's instructions (NZYTech, Lisbon, Portugal). To confirm nongenomic DNA synthesis a standard end‐point PCR for β‐actin, using intron‐spanning primers (Table 2), was performed with 2× MyTaq Red Mix (Bioline, London, UK), as previously described (Santiago et al., 2009). The cDNA samples were diluted 1:2 and kept at −20°C until further analysis.

**Table 2:** List of primers used in this work

Gene	RefSeq accession	Forward	Reverse	Amplicon size
*Adora2a*	NM_009630	5′‐TCCTGCTAATACACTACTCTC‐3′	3′‐TCCTCACATTGTTATCTTCTTG‐5′	107 bp
*Adora2a*	NM_053294	5′‐GGCTATCTCTGACCAACA‐3′	3′‐TGGCTTGACATCTCTAATCT‐5′	106 bp
*Itgam*	NM_012711	5′‐AAGGTCATACAGCATCAGT‐3′	3′‐GTTGATCTGGACAGGGAT‐5′	106 bp
*Tspo*	NM_012515.2	5′‐TGTATTCGGCCATGGGGTATG‐3′	3′‐GAGCCAGCTGACCAGTGTAG‐5′	105 bp
*Cd74*	NM_013069.2	5′‐CCACCTAAAGAGCCACTGGA‐3′	3′‐AGAGCTGGCTTCTGTCTTCAC‐5′	101 bp
*Il1b*	NM_031512	5′‐CTGTCTGACCCATGTGAG‐3′	3′‐TTGTCGTTGCTTGTCTCT‐5′	107 bp
*Tnf*	NM_012675	5′‐CCCAATCTGTGTCCTTCT‐3′	3′‐TTCTGAGCATCGTAGTTGT‐5′	90 bp
*Trem2*	NM_001106884.1	5′‐AACTTCAGATCCTCACTGGACC‐3′	3′‐CCTGGCTGGACTTAAGCTGT‐5′	90 bp
*Hprt1*	XM_003752155	5′‐ATGGGAGGCCATCACATTGT‐3′	3′‐ATGTAATCCAGCAGGTCAGCAA‐5′	77 bp
*Ywhaz*	NM_013011.3	5′‐CAAGCATACCAAGAAGCATTTGA‐3′	3′‐GGGCCAGACCCAGTCTGA‐5′	76 bp
*Tbp*	NM_001004198.1	5′‐ACCAGAACAACAGCCTTCCACCTT‐3′	3′‐TGGAGTAAGCCCTGTGCCGTAAG‐5′	116 bp
*Actb*	NM_031144.2	5′‐GCTCCTCCTGAGCGCAAG‐3′	3′‐CATCTGCTGGAAGGTGGACA‐5′	75 bp

The mRNA expression levels were quantified by quantitative polymerase chain reaction (qPCR) in a StepOnePlus™ Real‐Time PCR System (Applied Biosystems, Life Technologies, Carlsbad, CA) using a mix of 2 μL of 1:2 cDNA, 200 nM primers (Table 2; Sigma‐Aldrich, St. Louis, MO) and 20 μL of iTaq Universal SYBR Green Supermix (Bio‐Rad, Hercules, CA). Ct values were transformed into “relative quantification” using the 2^−ΔΔCt^ methods (Livak & Schmittgen, 2001). Three housekeeping candidate genes were tested: tyrosine 3‐monooxygenase/tryptophan 5‐monooxygenase activation protein, zeta polypeptide (ywhaz), hypoxanthine phosphoribosyltransferase 1 (hprt1), and TATA box binding protein (tbp). All samples were analyzed using NormFinder (Andersen, Jensen, & Orntoft, 2004), and the most stable gene among all samples and conditions was used as a housekeeping gene. In our conditions, ywhaz was the most stable gene and was used as a housekeeping gene.

### 2.19 Enzyme‐linked immunosorbent assay

The quantification of IL‐1β and TNF in the culture medium was performed by enzyme‐linked immunosorbent assay (ELISA), in accordance with the manufacturer's instructions (PeproTech, London, UK). Culture supernatants were collected and kept at −80°C until further analysis. Readings of the optical density were collected for 2 hr in 5 min intervals at 405 nm with wavelength correction set at 650 nm, using a microplate reader (Synergy HT; Biotek, Winooski, VT).

### 2.20 Statistical analysis

Results are presented as mean ± *SEM*. Shapiro–Wilk normality test was used to assess the normality of the data. Statistical analysis was performed using GraphPad Prism software version 6.01 for Windows (RRID:SCR_015807). For data with non‐Gaussian distribution, statistical significance was determined using nonparametric Kruskal–Wallis test followed by Dunn's multiple comparison tests or with nonparametric Mann–Whitney test; for data with Gaussian distribution, statistical significance was determined with parametric one‐way anova followed by Sidak's multiple comparisons test, as indicated in the figure legends. Results were considered statistically significant for *p* < 0.05.

## 3 RESULTS

### 3.1 EHP increases A_2A_R expression in BV‐2 microglia and primary retinal neural cell cultures

**Figure 1:**
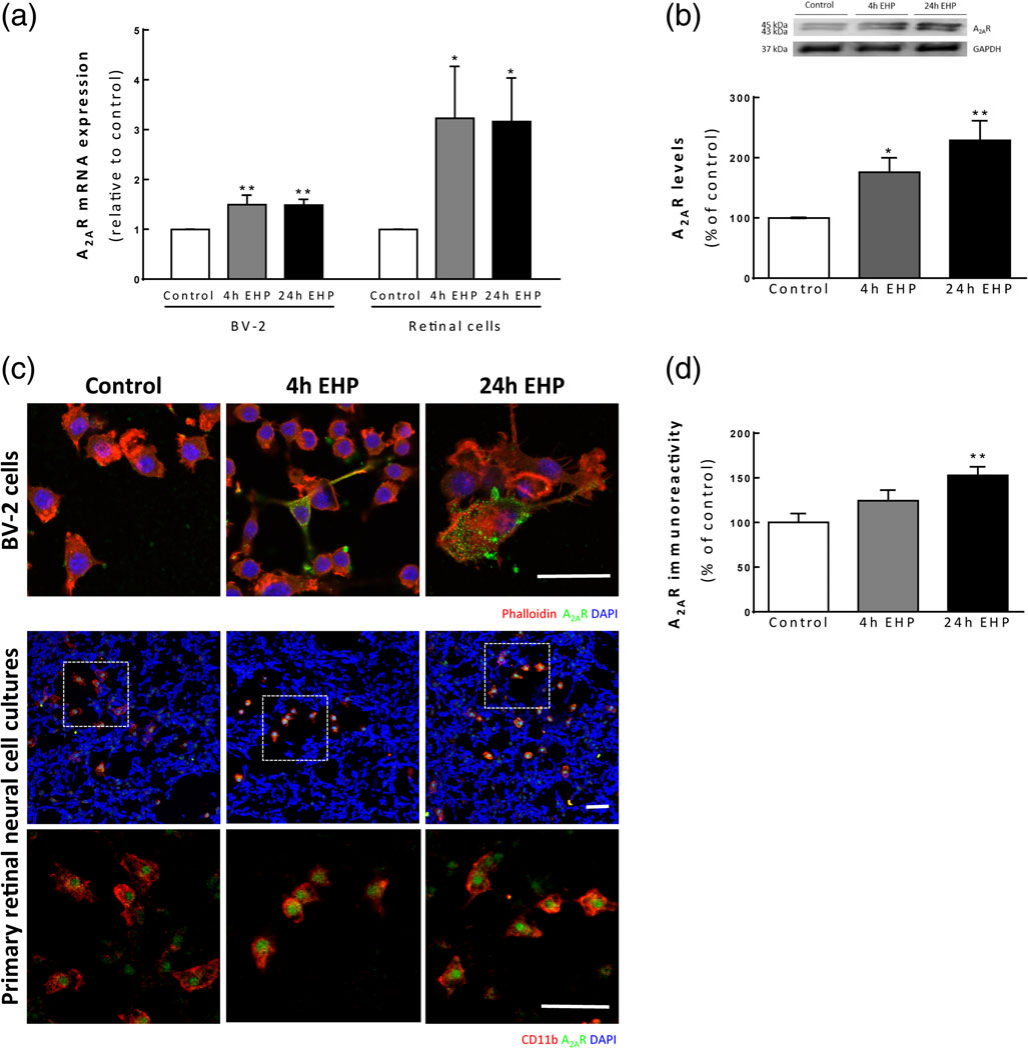
A_2A_R expression increases upon exposure to EHP in BV‐2 microglia and primary retinal neural cell cultures. (a) The A_2A_R mRNA expression was assessed in BV‐2 microglial cells and primary retinal cell cultures by qPCR and is presented as fold change of the control *n* = 4–5 and *n* = 8–11, respectively. (b) A_2A_R protein levels were assessed by Western blot in BV‐2 extracts. The densitometry of each band for A_2A_R was normalized for GAPDH, and the results are expressed as percentage of the control, *n* = 5–6. (c) A_2A_R immunoreactivity was assessed in BV‐2 and retinal cultures by immunocytochemistry. Representative images of BV‐2 cells stained with phalloidin (red) and microglia labeled with anti‐CD11b (red) in primary retinal cultures and anti‐A_2A_R (green) (c). Nuclei were stained with DAPI (blue). (d) In primary retinal neural cell cultures, the immunoreactivity of A_2A_R in CD11b^+^ cells was quantified, and is presented as percentage of the control, *n* = 3–4. *p < 0.05, **p < 0.01, compared with the control; Kruskal–Wallis test, followed by Dunn's multiple comparison test. Scale bar: 50 μm [Color figure can be viewed at wileyonlinelibrary.com]

BV‐2 microglia and primary retinal neural cells were challenged with EHP for 4 hr or 24 hr and the mRNA expression and protein levels of A_2A_R were assessed (Figure 1). The exposure of both cell cultures to EHP significantly increased the A_2A_R mRNA expression (1.5 ± 0.2 and 1.5 ± 0.1‐fold for BV‐2 cells (*p* < 0.01) and 3.2 ± 1.0 and 3.2 ± 0.9 fold for primary cultures (*p* < 0.05), at 4 and 24 hr of EHP exposure, respectively; Figure 1a). Consistent with the increase in the mRNA levels, we found an increase in A_2A_R protein levels in BV‐2 cells, as determined by Western blot (Figure 1b). The A_2A_R immunoreactivity was also assessed in BV‐2 cells and in primary retinal cultures by immunocytochemistry. The A_2A_R immunoreactivity increased in BV‐2 cells when exposed to EHP (Figure 1c). Retinal neural cell cultures are composed of neurons, astrocytes, Müller cells, and microglia (Santiago et al., 2007; Santos‐Carvalho, Elvas, Alvaro, Ambrósio, & Cavadas, 2013). In these cultures, all (100%) CD11b‐immunoreactive microglial cells express A_2A_R (Figure 1c). This observation was confirmed by the omission of the antibody anti‐CD11b, in which cells immunoreactive to A_2A_R have morphology consistent with microglia (Supporting Information Figure S1), faint labeling was also observed in other cells of the culture (Figure 1c). The exposure to EHP increased A_2A_R immunoreactivity in CD11b^+^ cells (Figure 1d).

### 3.2 A_2A_R pharmacological blockade or genetic deletion prevents EHP‐elicited increase in microglia migration and phagocytic efficiency

**Figure 2:**
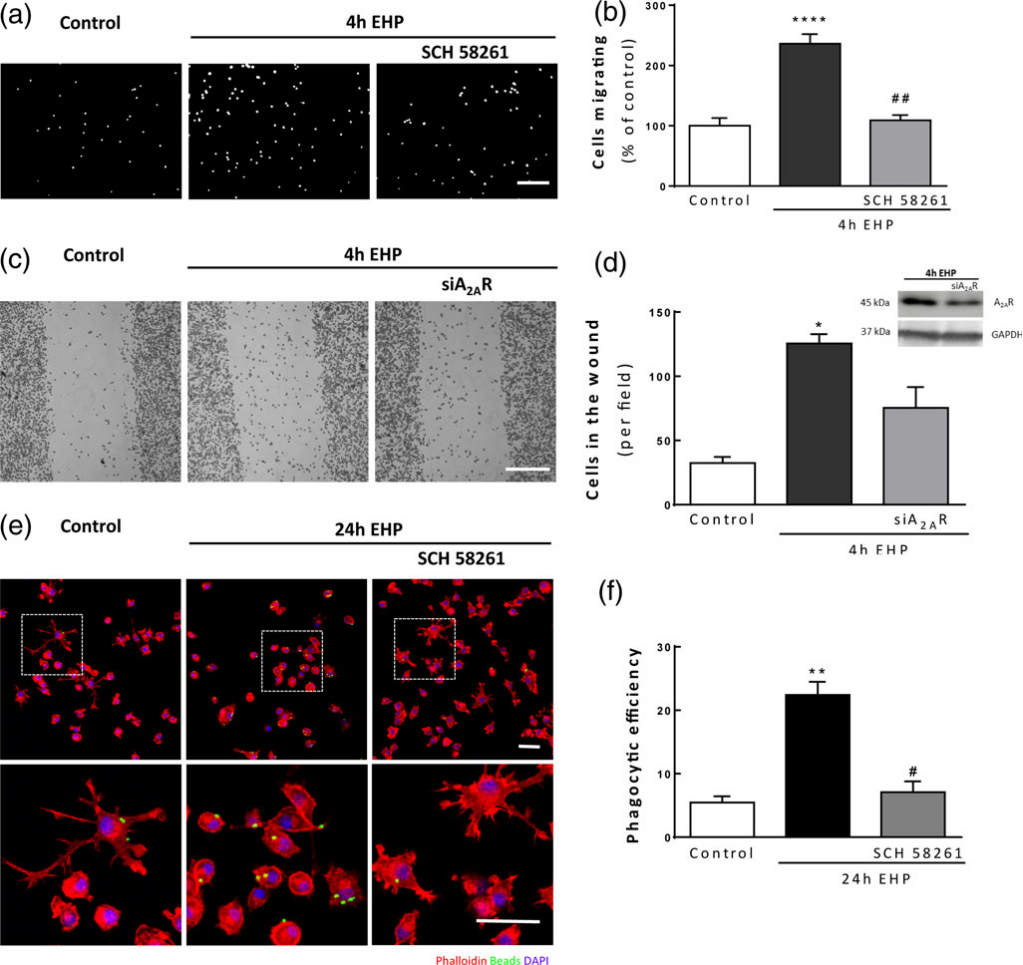
Gene silencing or pharmacologic blockade of A_2A_R inhibits the EHP‐induced increase in microglia motility and phagocytosis. Microglial cells were pretreated with 50 nM SCH 58261 or A_2A_R siRNA followed by exposure to EHP for 4 hr. BV‐2 microglial cell motility was assessed by the Boyden chamber migration (a and b) and scratch wound (c and d) assays. (b) The results are expressed as percentage of the control, *n* = 4–5. (d) The number of cells in the wound per field was counted, *n* = 3. In every experiment with siRNA A_2A_R, the decrease in the protein levels was confirmed by Western blot. (e) Phagocytosis was assessed in BV‐2 cells exposed to EHP for 24 hr using fluorescent beads. Representative images of BV‐2 cells stained with phalloidin (red) with incorporated beads (green). Nuclei were counterstained with DAPI (blue). (f) The phagocytic efficiency was calculated, *n* = 4–6. *p < 0.05, **p < 0.01, ****p < 0.0001, compared with control; #*p* < 0.05, ##*p* < 0.01, compared with EHP; Kruskal–Wallis test, followed by Dunn's multiple comparison test. Scale bar: 50 μm [Color figure can be viewed at wileyonlinelibrary.com]

Taking into consideration that EHP increased A_2A_R density in microglia, we evaluated whether impeding A_2A_R signaling could affect the motility of microglia. BV‐2 microglial cells were exposed to EHP for 4 hr and migration was assessed with the modified Boyden chamber assay (Figure 2a and b) and the scratch wound assay (Figure 2c and d). The exposure to EHP for 4 hr significantly increased the number of cells that migrate to the bottom side of the transwell insert (236.0 ± 16.1% of the control; *p* < 0.0001). This increase was abolished by the A_2A_R antagonist (109.2 ± 8.7% of the control; *p* < 0.01).

To confirm the results obtained with the A_2A_R antagonist in the modulation of EHP‐induced microglia migration, we used siRNA to knockdown A_2A_R in BV‐2 cells. The expression of A_2A_R was attenuated by 45.7% in EHP‐exposed BV‐2 cells with siRNA (Figure 2d). The genetic deletion of A_2A_R attenuated the EHP‐induced increase in microglia migration (from 399.5 ± 44.0% of the control in EHP conditions to 243.2 ± 57.6% in EHP + A_2A_R siRNA; Figure 2c and d), indicating that A_2A_R modulates microglial behavior when these cells are exposed to elevated pressure.

The phagocytic efficiency of BV‐2 microglia was determined after incubating the cells with fluorescent latex beads (Figure 2e and f). We observed that in control conditions the majority of microglia did not have beads inside. In cells exposed to EHP for 24 hr, there was a significant increase in the number of beads phagocytized (Phago Eff. = 22.4 ± 2.1%; *p* < 0.01) when compared with control (Phago Eff. = 5.5 ± 1.0%). The incubation with the A_2A_R antagonist prevented the increase in phagocytic efficiency induced by EHP (Phago Eff. = 7.1 ± 1.7%; *p* < 0.05).

### 3.3 Blockade of A_2A_R prevents the response of primary retinal microglia to EHP

**Figure 3:**
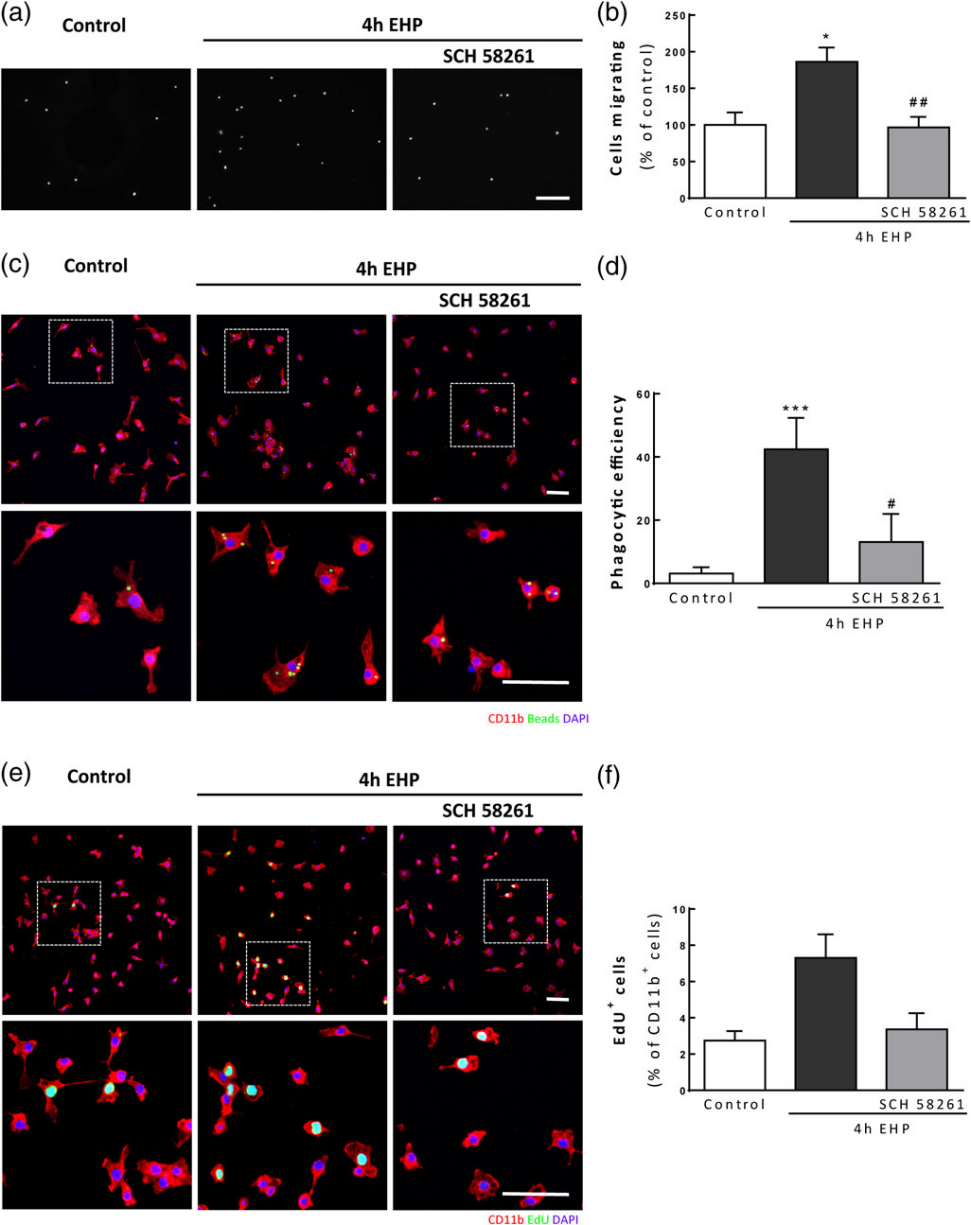
Blockade of A_2A_R in primary retinal microglia controls cell response to EHP. Primary retinal microglial cells were pretreated with 50 nM SCH 58261 and then exposed to EHP for 4 hr. Cell motility was assessed by the Boyden chamber assay (a and b). The results are presented as percentage of control (b), *n* = 6. (c and d) Phagocytosis was assessed in primary retinal microglial cells exposed to EHP for 4 hr using fluorescent beads. (c) Representative images of CD11b^+^ cells (red) with incorporated beads (green). Nuclei were counterstained with DAPI (blue). (d) The phagocytic efficiency was calculated, *n* = 6. (e) Representative images of microglia labeled with anti‐CD11b (red) and stained with EdU (green). Nuclei were stained with DAPI (blue). (f) The number of proliferating microglia (EdU^+^ CD11b^+^ cells) was counted and expressed as percentage of total microglia. *p < 0.05, ***p < 0.001, compared with control; #*p* < 0.05, ##*p* < 0.01, compared with EHP; Kruskal–Wallis test, followed by Dunn's multiple comparison test. Scale bar: 50 μm [Color figure can be viewed at wileyonlinelibrary.com]

Primary retinal microglia were pretreated with the A_2A_R antagonist and exposed to EHP for 4 hr. Microglial cell migration was evaluated by the Boyden chamber migration assay (Figure 3a). The exposure to EHP increased cell motility (186.1 ± 19.5% of the control, *p* < 0.05) that was prevented by the incubation with SCH 58261 (96.5 ± 14.3% of the control, *p* < 0.01; Figure 3b). Retinal microglia phagocytic efficiency was evaluated with latex beads (Figure 3c). The exposure to EHP increased the number of beads phagocytosed by microglia (Phago Eff. = 42.4 ± 4.1%; *p* < 0.001) when compared with control conditions (Phago Eff. = 3.1 ± 0.9%). Pretreatment with A_2A_R prevented the increase in microglia phagocytosis (Phago Eff. = 13.1 ± 3.6%; *p* < 0.05) (Figure 3d). Microglia proliferation was evaluated with EdU (Figure 3e and f), an analog of thymidine that intercalates in the DNA during S phase, and, therefore, a nuclear marker of cell division (Salic & Mitchison, 2008). Exposure to EHP increased the number of proliferating microglia (7.3 ± 1.3% of CD11b^+^ cells) when compared with control conditions (2.7 ± 0.5% of CD11b^+^ cells). Preincubation with SCH 58261 decreased the number of proliferating microglial cells (3.4 ± 0.9% of CD11b^+^ cells).

### 3.4 A_2A_R blockade hampers microglia proliferation and morphological changes in primary rat retinal cultures induced by EHP

**Figure 4:**
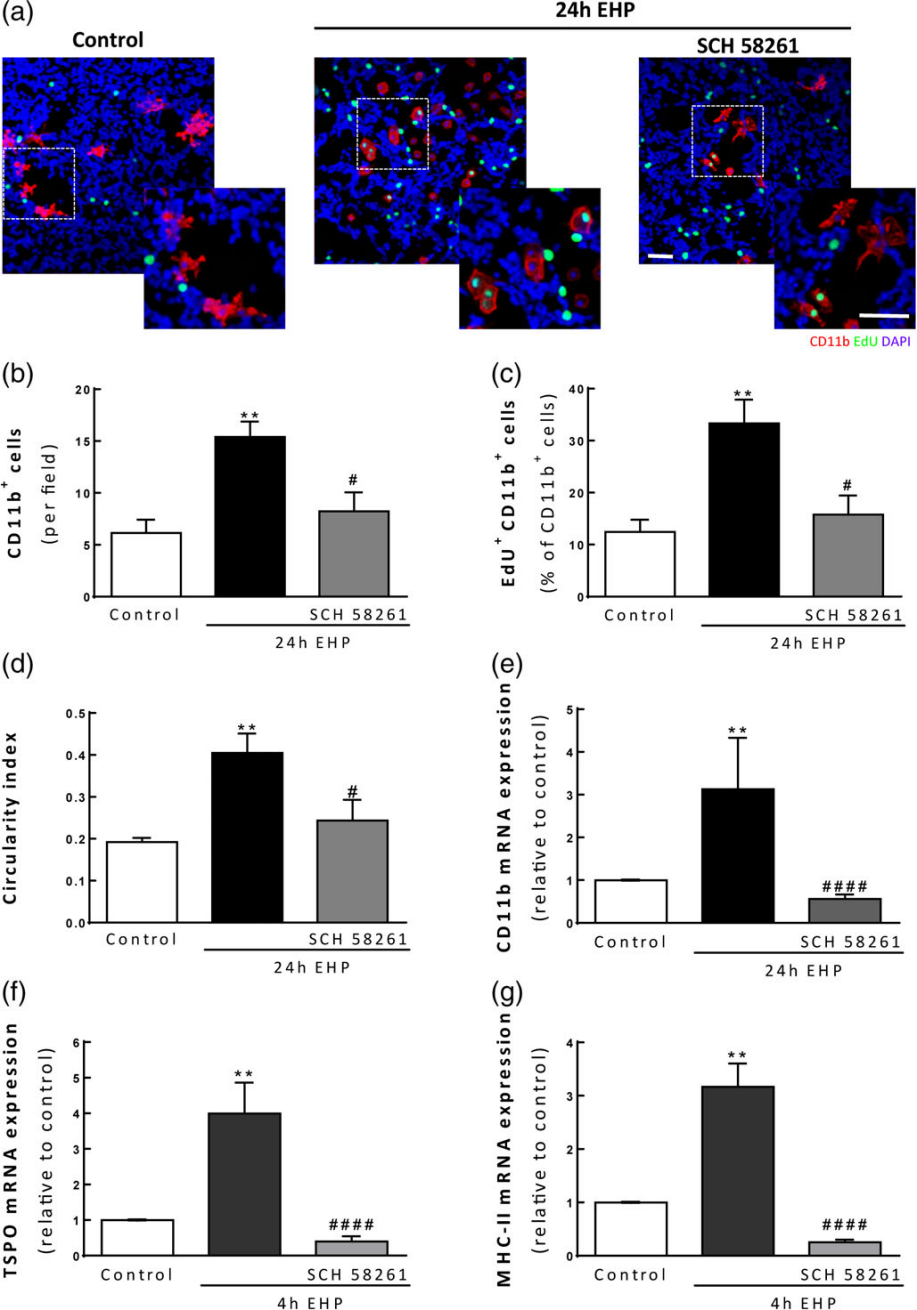
The antagonist of A_2A_R prevents EHP‐induced changes in microglia morphology and cell response. Primary retinal neural cell cultures were pretreated with 50 nM SCH 58261 following exposure to EHP for 24 hr. (a) Microglial cells were labeled using anti‐CD11b (red) and cell proliferation was measured by counting the number of EdU^+^ cells (green). Nuclei were counterstained with DAPI (blue). (b) The number of CD11b^+^ cells per field was counted, *n* = 6–7. (c) The number of microglial cells proliferating (EdU^+^CD11b^+^ cells) was counted and the results are expressed as the ratio EdU^+^CD11b^+^/CD11b^+^, *n* = 6–8. (d) The circularity index was determined using ImageJ, *n* = 6–8. The mRNA expression levels of CD11b (e), TSPO (f) and MHC II (g) were assessed by qPCR and the results are presented as fold change of the control, *n* = 4–6. **p < 0.01, compared with control; #*p* < 0.05, ####*p* < 0.001, compared with EHP; Kruskal–Wallis test, followed by Dunn's multiple comparison test. Scale bar: 50 μm [Color figure can be viewed at wileyonlinelibrary.com]

Given that blocking the actions of A_2A_R prevented the effects of EHP on migration and phagocytosis in BV‐2 microglial cells and on migration, phagocytosis, and proliferation in primary retinal microglia, we further investigated whether the blockade of A_2A_R could also modulate the response of microglia to EHP in primary retinal neural cell cultures. The exposure to EHP for 24 hr significantly increased the number of microglial cells in culture (15 ± 2 CD11b^+^ cells/field; *p* < 0.01) comparing with the control (6 ± 1 CD11b^+^ cells/field) and the blockade of the A_2A_R prevented this effect (8 ± 2 CD11b^+^ cells/field; *p* < 0.05; Figure 4a and b). In order to assess if the increase in microglial cell number was due to cell proliferation, we performed a proliferation assay (Figure 4a and c). In control conditions, only 12.4 ± 2.4% of the total CD11b^+^ cells were proliferating whereas the exposure to EHP for 24 hr lead to a 2.7‐fold increase in microglia proliferation (33.3 ± 4.6% of the total CD11b^+^ cells; *p* < 0.01). The A_2A_R antagonist prevented the proliferation of microglia elicited by EHP (15.8 ± 3.7% of CD11b^+^ cells; *p* < 0.05). Non‐CD11b^+^ cells also incorporated EdU, indicating that apart from microglia, other cells in the primary rat retinal cultures are also proliferating.

We also assessed the circularity index (CI) of microglial cells, which can be used as a measurement of the microglia morphology (Figure 4d). Microglia shifted from a more ramified morphology in control conditions (CI = 0.19 ± 0.01) toward a more amoeboid morphology when exposed to EHP for 24 hr (CI = 0.41 ± 0.05; *p* < 0.01). Incubation with SCH 58261 prevented the alterations in cell morphology (CI = 0.24 ± 0.05; *p* < 0.05) elicited by EHP.

Furthermore, A_2A_R blockade also prevented the effect of EHP exposure on the increase of mRNAs coding CD11b (Itgam), mitochondrial 18 kDa Translocator Protein (Tspo) and major histocompatibility complex class II family (MHC II, gene Cd74; Figure 4e–g).

### 3.5 Blockade of A_2A_R prevents oxidative stress and the release of pro‐inflammatory mediators triggered by EHP

**Figure 5:**
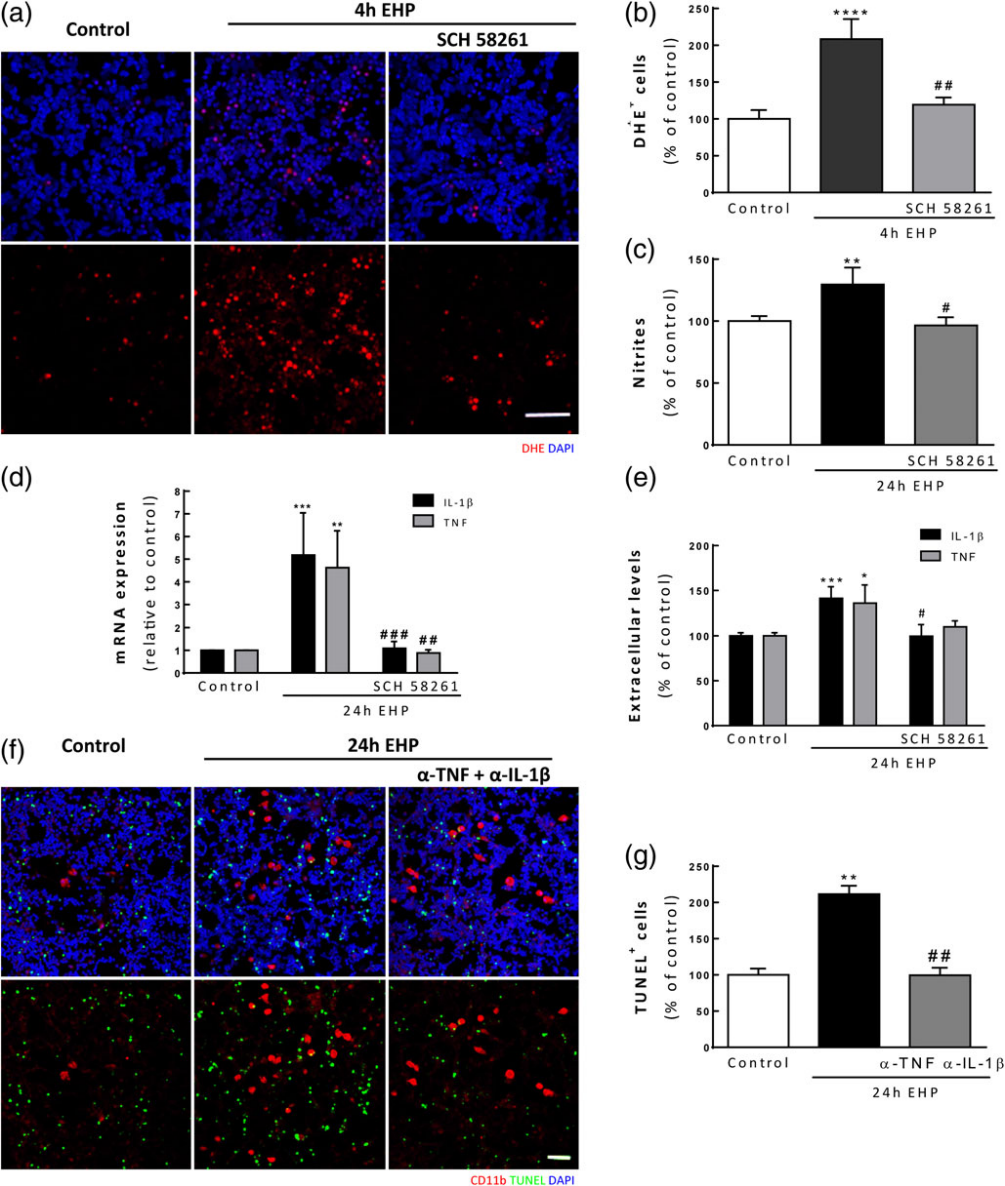
The blockade of A_2A_R prevents oxidative/nitrosative stress and the release of pro‐inflammatory mediators triggered by EHP in primary retinal neural cell cultures. (a) The production of ROS was assessed by DHE staining (red). Nuclei were stained with DAPI (blue). (b) The number of DHE^+^ was counted, and the results are expressed as percentage of the control, *n* = 5–9. (c) Extracellular nitrites concentration was quantified in supernatants from primary retinal neural cell cultures by the Griess reaction method, *n* = 6. (d) The mRNA expression levels of TNF and IL‐1β were assessed by qPCR, *n* = 7–10. (e) The protein levels of TNF and IL‐1β in culture supernatants of primary retinal neural cell cultures were determined by ELISA, *n* = 7–11. (f) Primary retinal neural cell cultures were pretreated with antibodies to neutralize the actions of TNF and IL‐1β and exposed to EHP for 24 hr. Cell death was assessed with TUNEL assay. The number of TUNEL^+^ cells (green) was counted (g). Nuclei were stained with DAPI (blue). *p < 0.05, **p < 0.01, ***p < 0.001, ****p < 0.0001, compared with control; #*p* < 0.05, ##*p* < 0.01, ###*p* < 0.001; compared with EHP, Kruskal–Wallis test, followed by Dunn's multiple comparison test (b, c, e, g) or one‐way anova followed by Sidak's multiple comparisons test (d). Scale bar: 50 μm [Color figure can be viewed at wileyonlinelibrary.com]

The impact of EHP on oxidative stress of retinal neural cells was assessed by evaluating DHE staining following exposure to EHP for 4 hr (Figure 5a). The exposure to EHP significantly increased the number of cells stained with DHE (208.4 ± 27.1% of the control; *p* < 0.0001; Figure 5b). The A_2A_R antagonist, SCH 58261, prevented the effect of EHP (119.2 ± 9.9% of the control; *p* < 0.01). The production of NO was determined by quantifying the formation of nitrites in cell culture medium by the Griess reaction method (Figure 5c). In control conditions, the nitrites concentration was 4.1 ± 0.2 μM and the exposure to EHP for 24 hr significantly increased nitrite concentration to 129.5 ± 13.7% of the control (*p* < 0.01). The blockade of A_2A_R prevented the effect of EHP on nitrite concentration (96.5 ± 6.6% of the control; *p* < 0.05).

We also evaluated the impact of EHP on the expression and release of the pro‐inflammatory cytokines IL‐1β and TNF (Figure 5d and e) from retinal neural cell cultures. The exposure of retinal cultures to EHP for 4 hr significantly increased the mRNA levels of IL‐1β and TNF (5.2 ± 1.9 and 4.6 ± 1.6‐fold above the control, for IL‐1β and TNF, respectively), and this effect was prevented by the blockade of A_2A_R. Moreover, the exposure of primary cultures to EHP for 24 hr significantly increased the extracellular levels of TNF (136.0 ± 20.3% of the control; *p* < 0.05) and IL‐1β (141.5 ± 12.8% of the control; *p* < 0.001) (Figure 5e). The blockade of A_2A_R prevented the increase in pro‐inflammatory cytokines expression and their release in primary retinal neural cell cultures (Figure 5d and e).

To unravel the importance of the pro‐inflammatory cytokines TNF and IL‐1β to retinal cell death, primary retinal neural cell cultures were pretreated with antibodies anti‐TNF and anti‐IL‐1β to neutralize the actions of these cytokines (Figure 5f and g). EHP significantly increased the number of TUNEL^+^ cells (211.5 ± 11.4% of the control; *p* < 0.01). The neutralization of TNF and IL‐1β actions prevented the increase in the number of TUNEL^+^ cells induced by EHP (99.5 ± 10.5% of the control; *p* < 0.01).

Overall, this group of results suggests that EHP triggers neural cell death mediated by TNF and IL‐1β and that the A_2A_R antagonist is able to prevent this effect probably by decreasing the levels of these pro‐inflammatory cytokines. In order to further confirm this hypothesis, we co‐incubated retinal neural cells with anti‐TNF, anti‐IL‐1β, and SCH 58261 prior the exposure to EHP and determined cell death. The neutralization of the actions of TNF and IL‐1β decreased the number of TUNEL^+^ cells induced by EHP, which was not significantly different when the cells were co‐incubated with SCH 58261 (Supporting Information Figure S2). This lack of additive effects indicates that the protective effects of A_2A_R antagonist in EHP conditions occur by the modulation of these pro‐inflammatory cytokines. Interestingly, when each antibody against the pro‐inflammatory mediators has incubated alone the effect on the reduction of cell death was not so pronounced (Supporting Information Figure S2).

### 3.6 A_2A_R blockade prevents EHP‐induced cell death and engulfment of dead cells by microglia

**Figure 6:**
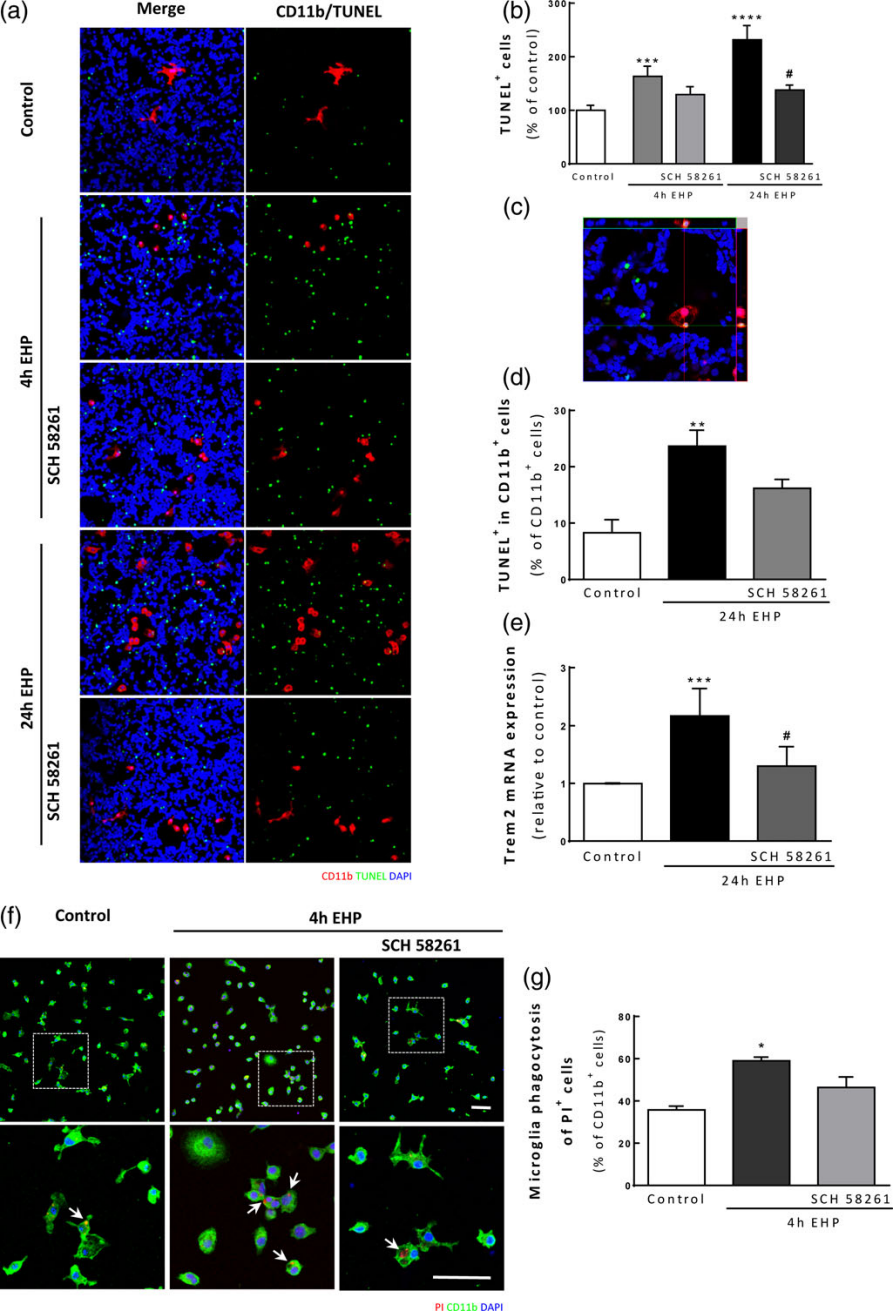
A_2A_R blockade prevents EHP‐induced cell death in primary retinal neural cell cultures and decreases dead/dying cell engulfment by primary microglia. (a) Cell death was assessed by TUNEL assay. Microglial cells were identified by immunocytochemistry with anti‐CD11b (red) and nuclei were counterstained with DAPI (blue). (b) The number of TUNEL^+^ cells (green) was counted and the results are expressed as percentage of the control *n* = 6–10. (c) Orthogonal confocal image representing microglial engulfment of a TUNEL^+^ cell. (d) The number of microglial cells with engulfed TUNEL^+^ cells (TUNEL^+^ in CD11b^+^ cells) was counted, and the results are expressed as the percentage of total microglial cells per field, *n* = 5–6. (e) The mRNA expression levels of Trem2 were assessed by qPCR, *n* = 5–6. (f) Phagocytosis of PI‐labeled retinal cells (red) by primary retinal microglia labeled with anti‐CD11b, green). Nuclei were counterstained with DAPI (blue). Arrows indicate some engulfed PI^+^ cells. (g) The number of microglia with engulfed PI^+^ cells was counted and presented as a percentage of total microglia, *n* = 4. *p < 0.05, **p < 0.01, ***p < 0.001, ****p < 0.0001, compared with control; #*p* < 0.05, compared with EHP; Kruskal–Wallis test, followed by Dunn's multiple comparison test. Scale bar: 50 μm [Color figure can be viewed at wileyonlinelibrary.com]

Exposure of retinal cells to EHP for 4 and 24 hr significantly increased the number of TUNEL^+^ cells to 163.4 ± 19.0% (*p* < 0.001) and 231.5 ± 26.7% (*p* < 0.0001) of the control, respectively. Treatment with SCH 58261 prevented the increase in cell death induced by EHP at 4 and 24 hr to 129.3 ± 14.8% and 137.8 ± 9.3% of the control, respectively (Figure 6a and b). Microglial cells (CD11b^+^) stained with TUNEL in the nucleus were rarely detected, indicating that EHP does not induce apoptosis of microglia. However, we observed that several microglial cells were positive for TUNEL in the cytoplasm (TUNEL^+^ in CD11b^+^ cells), indicating that microglia were phagocytizing apoptotic cells (Figure 6c). The number of microglia with engulfed TUNEL^+^ cells was significantly increased upon exposure to EHP for 24 hr (23.7 ± 2.8 TUNEL^+^ in CD11b^+^ cells per field; *p* < 0.01) comparing with control (8.3 ± 2.3 TUNEL^+^ in CD11b^+^ % of CD11b^+^ cells). The incubation with the A_2A_R antagonist decreased the number of microglia with engulfed dead cells (16.2 ± 1.6 TUNEL^+^ in CD11b^+^ cells per field; Figure 6d).

The expression of triggering receptor expressed on myeloid cells 2 (Trem2) has been associated with phagocytosis of apoptotic neuronal cells (Hsieh et al., 2009; Kawabori et al., 2015). The mRNA levels of Trem2 increased by 2.2 ± 0.5‐fold in cultured retinal cells exposed to EHP (*p* < 0.001; Figure 6e), when compared with the control. The incubation with SCH 58261 decreased the expression of Trem2 (1.3 ± 0.3‐fold change of the control; *p* < 0.05). Then, to elucidate whether the effect of A_2A_R blockade on the decrease of phagocytosis (Figure 3c) was masked by the decrease in cell death (which would explain less engulfed cells), primary retinal microglial cells were exposed to dead retinal cells, previously stained with PI (Figure 6f and g). EHP increased the number of PI^+^ cells engulfed by microglia (59.0 ± 1.8% of total microglia; *p* < 0.05) when compared with control conditions (35.8 ± 1.8% of total microglia). The blockade of A_2A_R reduced the number of PI^+^ cells engulfed by microglia (46.4 ± 5.0% of total microglia). Taken together, these results show that EHP triggers neural apoptosis and renders microglia more prone to phagocytosis. In addition, blocking the actions mediated by A_2A_R signaling prevents neural cell death and microglia phagocytosis induced by EHP.

### 3.7 Microglia depletion prevents EHP‐induced cell death in primary retinal neural cell cultures

**Figure 7:**
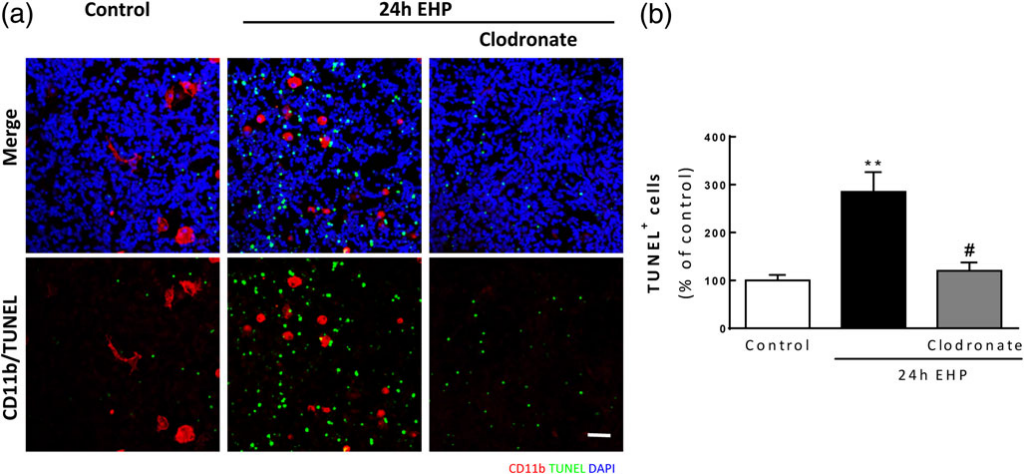
Microglia depletion prevents EHP‐induced cell death in primary retinal neural cell cultures. Microglial cells were depleted from primary retinal neural cell cultures using clodronate liposomes and then were exposed to EHP for 24 hr (a). Cell death was assessed by TUNEL assay (a and b). Microglial cells were identified by immunocytochemistry with anti‐CD11b (red) (a). Nuclei were counterstained with DAPI (blue). (b) The number of TUNEL^+^ cells (green) was counted, *n* = 4–8. **p < 0.01, compared with control; #*p* < 0.05, compared with EHP; Kruskal–Wallis, followed by Dunn's multiple comparison test. Scale bar: 50 μm [Color figure can be viewed at wileyonlinelibrary.com]

In order to assess the role of microglia in neural cell death in EHP conditions, microglia were depleted from the cultures with clodronate‐loaded liposomes (Kumamaru et al., 2012). The incubation with clodronate liposomes for 24 hr was able to eliminate microglia, as observed by the absence of CD11b^+^ cells (Figure 7a). The depletion of microglia clearly decreased the number of TUNEL^+^ cells (120.2 ± 17.5% of the control; *p* < 0.05) when compared with cultures exposed 24 hr to EHP (284.9 ± 41.1% of the control; *p* < 0.01). In addition, when the cultures were incubated with 25 nM PLX3397, an inhibitor of colony stimulating factor 1 receptor (Elmore et al., 2014), thus decreasing the number of microglial cells under EHP to 41.9 ± 2.2% of the control, the EHP‐induced cell death was prevented (Supporting Information Figure S3). These results strongly suggest that microglia contribute to the neural cell loss under EHP conditions.

In order to establish whether microglial cells are the source of TNF and IL‐1β under EHP conditions, the concentration of these cytokines was measured in supernatants of microglia‐depleted cultures (Supporting Information Figure S4). The levels of TNF were maintained elevated in the clodronate‐treated cultures exposed to EHP (Supporting Information Figure S4A), while the levels of IL‐1β decreased to control levels (Supporting Information Figure S4B). These results indicate that in these cultures when exposed to EHP, microglia release IL‐1β but not TNF.

### 3.8 A_2A_R antagonist decreases microglia response to EHP in human organotypic retinal cultures

**Figure 8:**
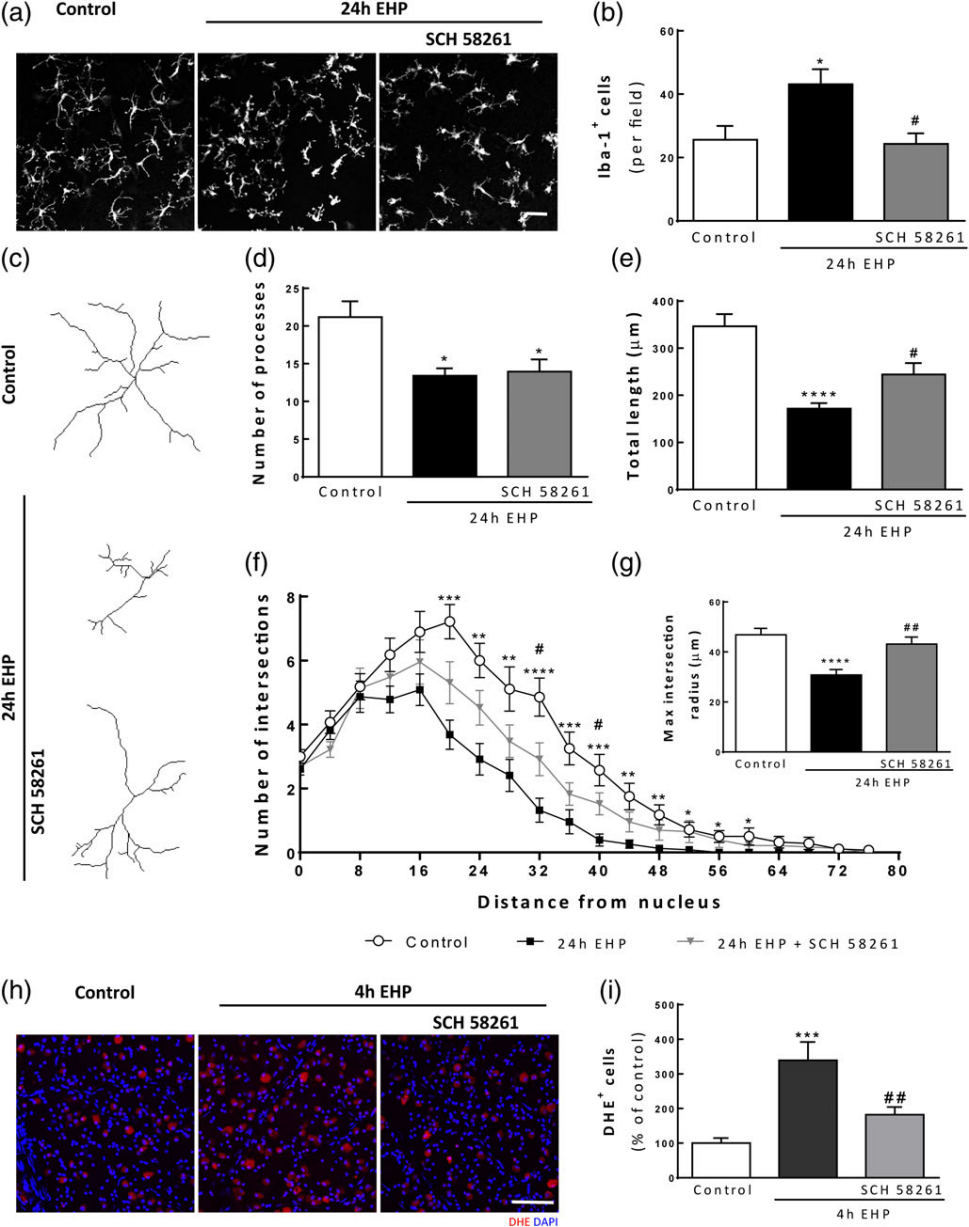
The blockade of A_2A_R inhibits microglial cell response in human organotypic retinal cultures. Human retinal explants were pretreated with 50 nM SCH 58261 followed by exposure to EHP for 4 hr or 24 hr. (a) Microglial cells were labeled by immunocytochemistry with an antibody anti‐Iba‐1 (white). (b) The number of Iba‐1^+^ cells per field was counted, *n* = 5. Microglia morphologic features were assessed from 3D reconstructed images (c). The total number of processes (d), total length (e), Sholl analysis (f), and last intersection radius (g) were analyzed from four independent donors in a total of 21–28 cells analyzed per condition. Results represent the average morphologic features from the total number of cells analyzed. ROS production was assessed by DHE staining (h). Nuclei were stained with DAPI (blue). (i) The number of DHE^+^ cells (red) was counted *n* = 6–7. *p < 0.05, ***p < 0.001, ****p < 0.0001, compared with control; #*p* < 0.05, ##*p* < 0.01, compared with EHP; Kruskal–Wallis, followed by Dunn's multiple comparison test (b, d, and f) or one‐way anova followed by Sidak's multiple comparisons test (e, g, and i). Scale bar: 50 μm [Color figure can be viewed at wileyonlinelibrary.com]

Taking into account the results obtained in BV‐2 cell line, primary retinal microglial and primary retinal neural cell cultures, we also aimed to assess whether the A_2A_R antagonist had the ability to modulate the response of microglia to EHP in human retinas. Human retinal organotypic cultures were pretreated with 50 nM SCH 58261 and exposed to EHP for 24 hr. The number of microglial cells was assessed by counting the number of cells immunoreactive to Iba‐1 (ionized calcium‐binding adapter molecule 1; Figure 8a and b). The number of microglial cells increased in human organotypic retinal cultures exposed to EHP (43 ± 5 Iba1^+^ cells; *p* < 0.05), compared with the control (26 ± 4 Iba1^+^ cells). Pretreatment with SCH 58261 prevented the increase in the number of microglial cells (24 ± 3 Iba1^+^ cells; *p* < 0.05).

Furthermore, human microglia morphology was greatly impacted by EHP. The total number of processes of microglial cells decreased from 21 ± 2 in the control to 13 ± 1 in EHP conditions (Figure 8c and d; *p* < 0.05) and the total length of each cell decreased from 346.7 ± 26.0 μm in the control to 171.8 ± 11.6 μm in EHP conditions (Figure 8e; *p* < 0.001), indicating that human microglia in EHP are smaller and less ramified. Treatment with SCH 58261 did not change the effect of EHP in the total number of processes (Figure 8d) but inhibited the decrease in the total length of microglia (244.0 ± 24.5 μm; *p* < 0.05). The analysis of microglia arborization was performed by Sholl analysis (Figure 8f and g). Human microglia exposed to EHP presented a reduction in branching complexity as a function of distance from the nucleus compared with control, which was slightly reestablished by the treatment with SCH 58261. The maximum intersection radius (extracted from the Sholl analysis), which provides an estimation of the size of the “territory” occupied by microglia, revealed a 1.6 decrease in microglia last intersection, indicating that human microglia exposed to EHP are less complex. Treatment with SCH 58261 prevented the decrease in microglia arborization, as shown by the increase in the maximum intersection radius (from 30.8 ± 2.2 μm in EHP to 43.1 ± 2.9 μm in EHP with SCH 58261; *p* < 0.05).

We then addressed if elevated pressure increased oxidative stress in human organotypic cultures and if A_2A_R blockade was able to prevent this effect. The production of ROS was determined by DHE staining (Figure 8h and i). Exposure to EHP for 4 hr significantly increased the number of DHE^+^ cells in the ganglion cell layer (339.4 ± 53.1% of the control; *p* < 0.001) and the blockade of A_2A_R prevented the effect of EHP (182.1 ± 22.3% of the control; *p* < 0.01).

## 4 DISCUSSION

In the present study, we showed that microglial cells act as main players in retinal cell degeneration triggered by elevated pressure and unveiled the protective properties of microglial A_2A_R blockade. Moreover, this is the first study reporting that the A_2A_R selective antagonist prevents human retinal microglial cell response to elevated pressure.

Previous reports from us and others show that A_2A_R is expressed in the retina, including in microglia (Huang et al., 2014; Liou et al., 2008; Madeira, Elvas, et al., 2015; Vindeirinho, Costa, Correia, Cavadas, & Santos, 2013; Zhong, Yang, Huang, & Luo, 2013). The expression of A_2A_R has been demonstrated to be upregulated in brain chronic noxious conditions (Cunha, 2005; Vindeirinho et al., 2013; Wittendorp, Boddeke, & Biber, 2004). Elevated intraocular pressure is the main risk factor of glaucoma (Almasieh et al., 2012). In this work, cultures have been exposed to EHP in order to mimic elevated intraocular pressure in vitro (Aires et al., 2016). In our conditions, an increase in hydrostatic pressure induced upregulation of A_2A_R in microglia suggesting that microglia react to changes in pressure and A_2A_R may modulate the response of microglia to elevated pressure.

The blockade of A_2A_R confers neuroprotection in several models of neurodegeneration, including in the retina (Boia et al., 2016; Boia et al., 2017; Cunha, 2005; Gomes, Kaster, Tome, Agostinho, & Cunha, 2011; Gyoneva et al., 2014; Liu et al., 2016; Madeira, Boia, et al., 2016; Madeira, Elvas, et al., 2015). One of the mechanisms that may explain the protective properties of A_2A_R antagonists is the control of microglia‐mediated neuroinflammation (Cunha, 2005; Gomes et al., 2011). There is a controversy on the effects mediated by A_2A_R in pathological conditions, since A_2A_R activation in peripheral immune cells is anti‐inflammatory (Hasko & Cronstein, 2013; Hasko & Pacher, 2008; Sitkovsky & Ohta, 2005), and in chronic conditions of the central nervous system the blockade of the A_2A_R confers protection (Gomes et al., 2011). Therefore, we took advantage of our experimental models that lack infiltration of inflammatory cells from the periphery that also express A_2A_R to clarify the role of microglial A_2A_R in the protection of retinal cells against damage induced by elevated pressure.

Changes in microglia phenotype have been associated with the loss of RGCs in experimental and human glaucoma (Bosco et al., 2015; Bosco, Steele, & Vetter, 2011; Naskar, Wissing, & Thanos, 2002; Wang, Chen, Zhang, & Jonas, 2016; Yuan & Neufeld, 2001). Evidence shows that upon a noxious stimulus microglia become responsive and migrate toward the site of injury (Lourbopoulos, Erturk, & Hellal, 2015). ATP (Davalos et al., 2005; Dou et al., 2012; Gyoneva, Orr, & Traynelis, 2009; Imura et al., 2013), adenosine (Orr, Orr, Li, Gross, & Traynelis, 2009), and NO (Scheiblich et al., 2014) are key mediators for microglia mobilization. In this study, we found that the pharmacological blockade or genetic ablation of A_2A_R decreased microglia migration elicited by elevated pressure. Elevated pressure triggers an increase in extracellular levels of ATP and adenosine (Madeira, Elvas, et al., 2015; Rodrigues‐Neves et al., 2018), suggesting that ATP‐derived adenosine signals through A_2A_R that is upregulated in elevated pressure conditions, mediating microglia migration.

Adenosine through activation of the A_2A_R modulates microglia process retraction, inducing the amoeboid morphology characteristic of motile responsive microglial cells (Gyoneva et al., 2014; Koizumi, Ohsawa, Inoue, & Kohsaka, 2013; Orr et al., 2009). Indeed, alterations in microglia morphology in the retina and brain have been associated with inflammation and neuronal dysfunction (Bosco, Romero, Ambati, & Vetter, 2015; Davies, Ma, Jegathees, & Goldsbury, 2017; Morrison, Young, Qureshi, Rowe, & Lifshitz, 2017). When exposed to elevated pressure, microglial cells changed their morphology and became more amoeboid. It has been proposed that microglia motility and process retraction are orchestrated by receptor fluctuations and a concerted action of ATP in PY2 receptors and adenosine in A_2A_R (Koizumi et al., 2013). In this work, hampering the activity mediated by A_2A_R was sufficient to prevent the effects of elevated pressure in morphology and motility, probably by directly hindering microglia process retraction and therefore decreasing cell motility.

Another important function of microglia is the clearance of cell debris, dying and dead cells (Kettenmann, Kirchhoff, & Verkhratsky, 2013; Wolf, Boddeke, & Kettenmann, 2017). In chronic degenerative diseases, microglia become phagocytes with substantial deleterious effects for neurons or glial cells (Brown & Neher, 2014; Fu, Shen, Xu, Luo, & Tang, 2014; Napoli & Neumann, 2009). Recently, we demonstrated that blocking A_2A_R prevents LPS‐induced latex beads phagocytosis by retinal microglia (Madeira, Boia, et al., 2016). Herein, we found that the blockade of A_2A_R prevented an elevated pressure‐induced increase in the phagocytosis of beads by microglia (BV‐2 cell line and retinal primary microglia). Although LPS and elevated pressure may initiate the inflammatory cascade by acting on different pathways, the blockade of A_2A_R is sufficient to prevent microglial cell phagocytosis in both cases, suggesting some similarities between both insults. In the primary retinal neural cell cultures, the blockade of A_2A_R reduced both cell death and microglia phagocytosis. It is yet to be determined whether in these conditions, a decrease in phagocytosis is just a result of decreased cell death, which would reduce the number of cells to be engulfed by microglia, or if the A_2A_R antagonist acts on both mechanisms. Nevertheless, when primary retinal microglial cells were challenged with elevated pressure and exposed to dead retinal neural cells, an increase in the engulfment of dead cells was observed, indicating that elevated pressure triggers alterations in microglia, which become more prone to phagocytosis. The A_2A_R antagonist prevented this effect, suggesting that the response of microglia to elevated pressure is lost. Microglia proliferation has been described in several conditions (Davalos et al., 2005; de Gracia et al., 2015; Gomes et al., 2013; Gomez‐Nicola, Fransen, Suzzi, & Perry, 2013; Kettenmann, Hanisch, Noda, & Verkhratsky, 2011). Elevated pressure triggered microglia proliferation, indicating a switch from a surveillance status to an activated effector phenotype. Activation of A_2A_Rs in microglia has been shown to stimulate the proliferation of these cells, while the antagonist of A_2A_R was shown to hamper microglia proliferation upon an inflammatory stimulus (George et al., 2015; Gomes et al., 2013). In primary retinal neural cell cultures, proliferation was not exclusive to microglia. Astrocytes and Müller cells also play important roles in the inflammatory response, and pro‐inflammatory mediators increase their proliferation (de Hoz et al., 2016; Dyer & Cepko, 2000; Farina, Aloisi, & Meinl, 2007). Therefore, since these cells are also present in the culture, we cannot rule out the possibility that these cells are also proliferating (Bejarano‐Escobar, Sanchez‐Calderon, Otero‐Arenas, Martin‐Partido, & Francisco‐Morcillo, 2017). Retinal neuronal progenitor cells have also been identified in rat retinal cell cultures (Alvaro et al., 2008) and may also be proliferating. The blockade of A_2A_R prevented the increase in microglia number in retinal human and rat cultures, probably by regulating microglia proliferation, as was assessed in primary microglia. The effect in total cell proliferation can be due to the hindering of astrogliosis by A_2A_R antagonist (Dare, Schulte, Karovic, Hammarberg, & Fredholm, 2007), preventing astrocytic proliferation in response to the neuroinflammatory milieu brought by microglial cells.

Despite the putative role of other glial cells in the retina, it has been proposed that microglial cells are the major contributors for the neuroinflammatory response in several retinal diseases (Madeira, Boia, et al., 2015). Changes in microglia alertness state have been associated with increased expression of several proteins, namely, TSPO and MHC‐II (Jonas et al., 2012; Karlstetter et al., 2014; Ramírez et al., 2017; Scholz et al., 2015; Wang et al., 2014), and with a cytotoxic deleterious phenotype. Modeling ocular hypertension in vitro increased the expression of TSPO and MHC‐II in retinal neural cells, consistent with a response of microglia to elevated pressure. Also, A_2A_R antagonist prevented this increase, suggesting that microglial A_2A_R controls neuroinflammation, as has been suggested previously (Boia et al., 2017; Madeira et al., 2016).

The mechanisms underlying microglia alterations in glaucomatous optic neuropathy are not clarified yet. The increase in ROS has been described as an early event in the pathophysiology of neurodegenerative diseases, including glaucoma, perpetuating the activation of microglia and the release of pro‐inflammatory cytokines (Block & Hong, 2005; Block, Zecca, & Hong, 2007; Clausen et al., 2008; Liu & Hong, 2003; Lull & Block, 2010; Smith, Das, Ray, & Banik, 2012; Ye et al., 2016). IL‐1β and TNF are pro‐inflammatory cytokines produced by microglia that are involved in retinal neurodegeneration (Madeira, Boia, et al., 2015; Tezel & Wax, 2000; Wang et al., 2016; Yuan & Neufeld, 2001). Elevated pressure elicited a pro‐inflammatory environment, characterized by increased production of ROS, and the release of NO, IL‐1β, and TNF. In brain glial mixed cultures, the activation of A_2A_R increases ROS production by microglial cells (Saura et al., 2005). Contrarily, the blockade of A_2A_R prevented the formation of a pro‐inflammatory environment, decreasing the release of pro‐inflammatory mediators and reducing oxidative stress. Taking into consideration that similar results are observed in the four experimental models (microglia cell line, primary retinal microglia cultures, rat retinal neural cultures, and human tissue cultures), one can hypothesize that the effects are mediated by the A_2A_R present in microglia. The neutralization experiments using anti‐IL‐1β and anti‐TNF antibodies further corroborate the causal role of inflammation to retinal cell death. In our previous works (Madeira, Boia, et al., 2016; Madeira, Elvas, et al., 2015), we could not identify the cells releasing these cytokines, since other cells may participate in the inflammatory response (Chong & Martin, 2015; Soto & Howell, 2014; Wang, Cioffi, Cull, Dong, & Fortune, 2002; Yuan & Neufeld, 2000). In this work, we clearly demonstrate that microglia orchestrate an inflammatory response that is absolutely critical to damage retinal neurons. Interestingly, in the primary retinal neural cultures, we were able to identify microglia as the source of IL‐1β, but not TNF. In these cultures, TNF may be released by astrocytes and Müller cells, in the inflammatory response (Vargas & Di Polo, 2016; Yuan & Neufeld, 2000). This might be particular to the experimental conditions, where several cell types are present, since primary retinal microglia express TNF, when exposed to elevated pressure (Madeira, Boia, et al., 2016), despite at lower levels when compared with the expression of IL‐1β.

Elevated pressure increased neural cell death, and under these circumstances, apoptotic microglial cells were rarely observed. Although we cannot discard a direct impact of elevated pressure on neuronal function, since elevated pressure induces apoptosis in neuronal cell lines (Agar, Li, Agarwal, Coroneo, & Hill, 2006; Agar, Yip, Hill, & Coroneo, 2000), our findings show that elevated pressure changes microglia phenotype and these cells mount a pro‐inflammatory response that culminates in neuronal apoptosis. The essential role of microglia for neural apoptosis was further confirmed with the clodronate liposomes experiments. Under elevated pressure conditions, we detected microglia with engulfed dead/dying cells, as already observed in other disease models (Ferrer‐Martin et al., 2014; Jones et al., 1997; Petersen & Dailey, 2004). Microglia clearance of dead/dying cells is part of a mounted strategy to control the neuronal damage (Neumann, Kotter, & Franklin, 2009). Indeed, elevated pressure increases the phagocytosis by BV‐2 cells, without increasing BV‐2 cell death (data not shown). Taken together, these results indicate that the blockade of A_2A_R halts microglial cell response, thus inhibiting retinal cell death and reducing phagocytosis.

The potential anti‐inflammatory and protective effects of the A_2A_R antagonist were evaluated in human organotypic retinal cultures. SCH 58261 is also a potent and selective antagonist for human A_2A_R (Jones et al., 1997; Ongini, Dionisotti, Gessi, Irenius, & Fredholm, 1999) and it is suitable for in vitro pharmacological studies (Yang et al., 2007). In this model, we found that A_2A_R blockade by SCH 58261 was able to prevent microglia morphological alterations and ROS production triggered by elevated pressure, further reinforcing its role in the control of retinal neuroinflammation.

In summary, our results demonstrate that the blockade of microglial A_2A_R affords protection to retinal cells through the control of microglia response to damage, thus identifying microglial cells as major contributors for retinal cell death induced by elevated pressure. In addition, we also demonstrate that the A_2A_R antagonist prevents microglial cell response in the human retina. The A_2A_R emerges as an attractive target to manage retinal neuroinflammation in glaucoma.

## CONFLICT OF INTEREST

The authors declare no conflict of interest.

## Supporting information

Figure S1 Immunoreactivity of AZAR in primary retinal neural cell cultures. Primary retinal neural cell cultures were immunolabeled for AZAR. Negative control was performed by omitting the primary antibody. The labeling for AZAR alone (without anti‐CD11b) is depicted. Scale bar: 50 μm.

Figure S2 The AZAR antagonist lacks additive protective effects when co‐incubated with anti‐TNF and anti‐IL‐1B. Primary retinal neural cell cultures were pre‐treated with AZAR antagonist SCH 58261 and antibodies to neutralize the actions of TNF and IL‐1B and followed by exposure to EHP for 24 hr. Cell death was assessed by TUNEL assay and the number of TUNEL* cells (green) was counted, microglia were labeled with CD11b (red) for reference. Nuclei were stained with DAPI (blue). The results are expressed as percentage of the control from 1 to 4 independent experiments. Scale bar: 50 μm.

Figure S3 Reduction of microglia numbers in EHP conditions prevents the increase in cell death. The cells were incubated with 25 nM PLX3397 every day (from day 1 in culture until day 7). PLX3397, an inhibitor of colony‐stimulating factor‐1 receptor, used to deplete microglial cells (CD11b* cells, red) from the culture (a) prevented the increase in cell death (TUNEL* cells, green) induced by EHP (a and c). Nuclei were stained with DAPI (blue). The results are expressed as percentage of the control from three independent experiments. Scale bar: 50 μm. Number of microglial cell (Cd11b‐immunoreactive cells) in culture after PLX3397 incubation.

Figure S4 The depletion of microglia from primary retinal neural cell cultures exposed to EHP reduces the levels of IL‐1B but not TNF. Primary retinal neural cell cultures were depleted from microglia using clodronate liposomes and then exposed to EHP for 24 hr. The protein levels of TNF (a) and IL‐1B (b) in the supernatants were determined by ELISA. The results are presented in pg/mL from 3 to 4 (TNF) or 4 to 6 (IL‐1B) independent experiments. *p < 0.05, compared with control; Kruskal–Wallis test followed by Dunn's multiple comparison test.
