# Supplementary figures and images for: Blockade of microglial adenosine A2A receptor suppresses elevated pressure‐induced inflammation, oxidative stress, and cell death in retinal cells
Source: Glia. 2019 Jan 22;67(5):896–914. doi: 10.1002/glia.23579 (PMC6590475; doi:10.1002/glia.23579)

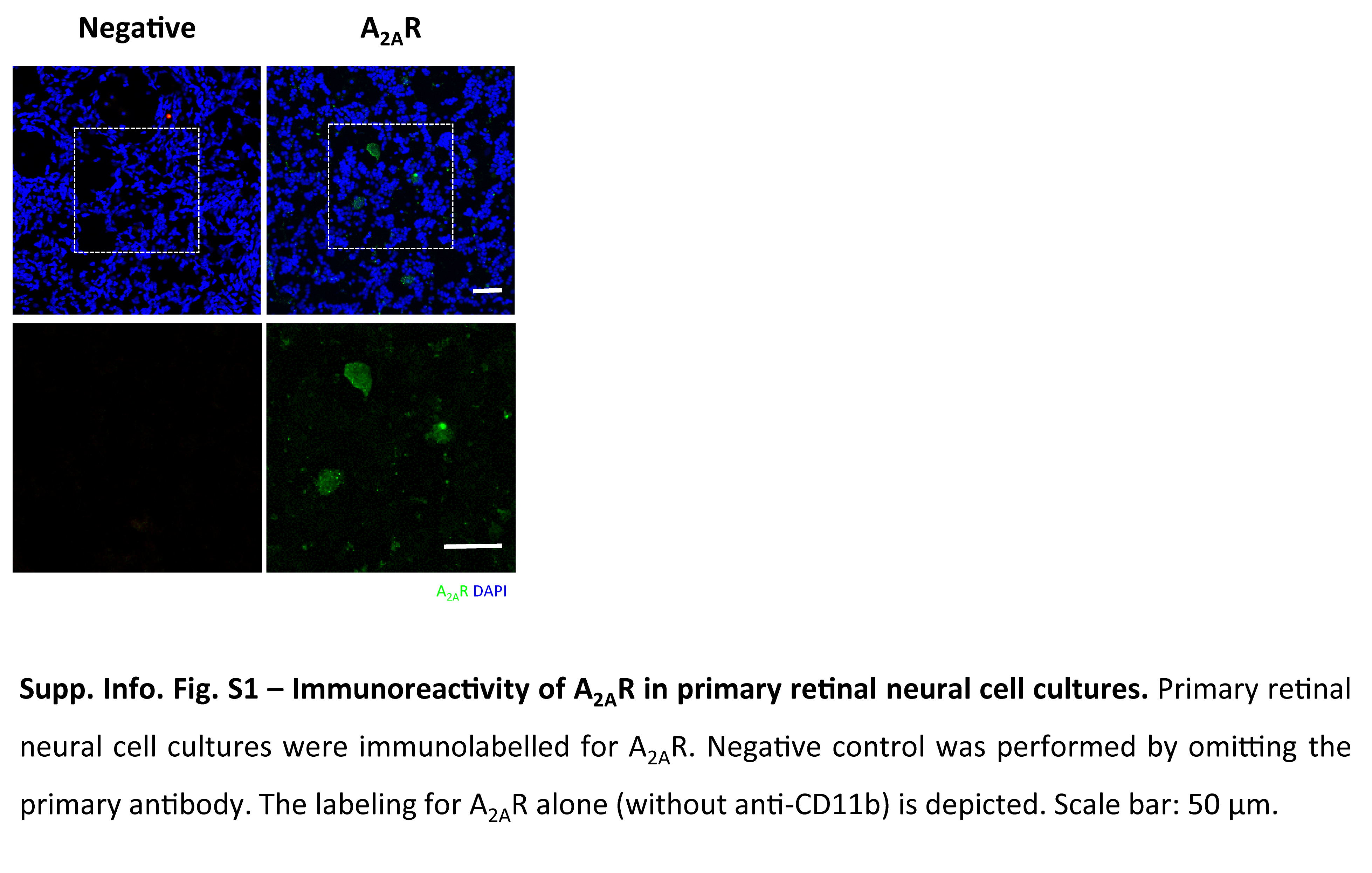

Supplement: Supplementary file 1 — Figure S1 Immunoreactivity of AZAR in primary retinal neural cell cultures. Primary retinal neural cell cultures were immunolabeled for AZAR. Negative control was performed by omitting the primary antibody. The labeling for AZAR alone (without anti‐CD11b) is depicted. Scale bar: 50 μm. [file GLIA-67-896-s001.tif]

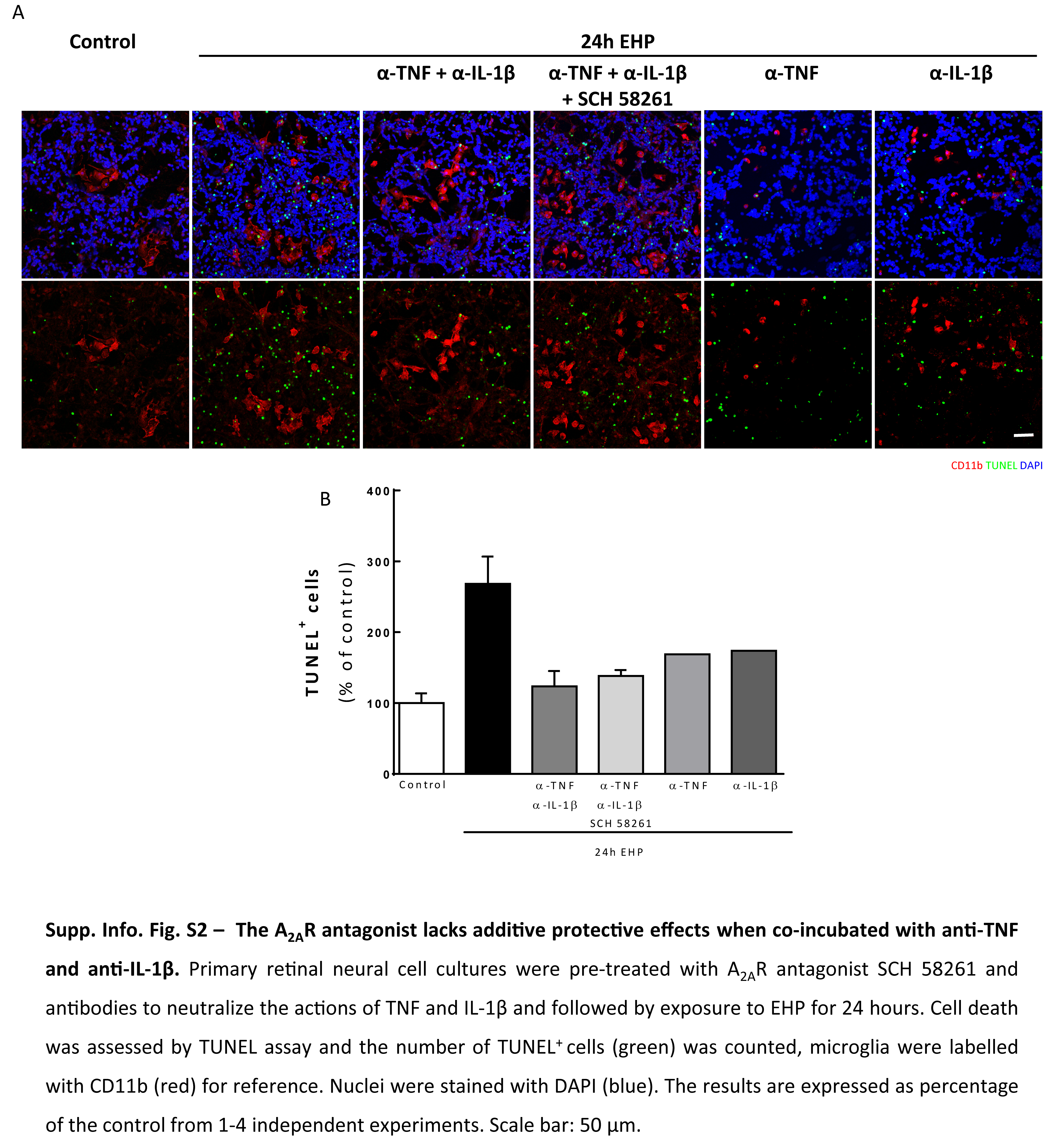

Supplement: Supplementary file 2 — Figure S2 The AZAR antagonist lacks additive protective effects when co‐incubated with anti‐TNF and anti‐IL‐1B. Primary retinal neural cell cultures were pre‐treated with AZAR antagonist SCH 58261 and antibodies to neutralize the actions of TNF and IL‐1B and followed by exposure to EHP for 24 hr. Cell death was assessed by TUNEL assay and the number of TUNEL* cells (green) was counted, microglia were labeled with CD11b (red) for reference. Nuclei were stained with DAPI (blue). The results are expressed as percentage of the control from 1 to 4 independent experiments. Scale bar: 50 μm. [file GLIA-67-896-s002.tif]

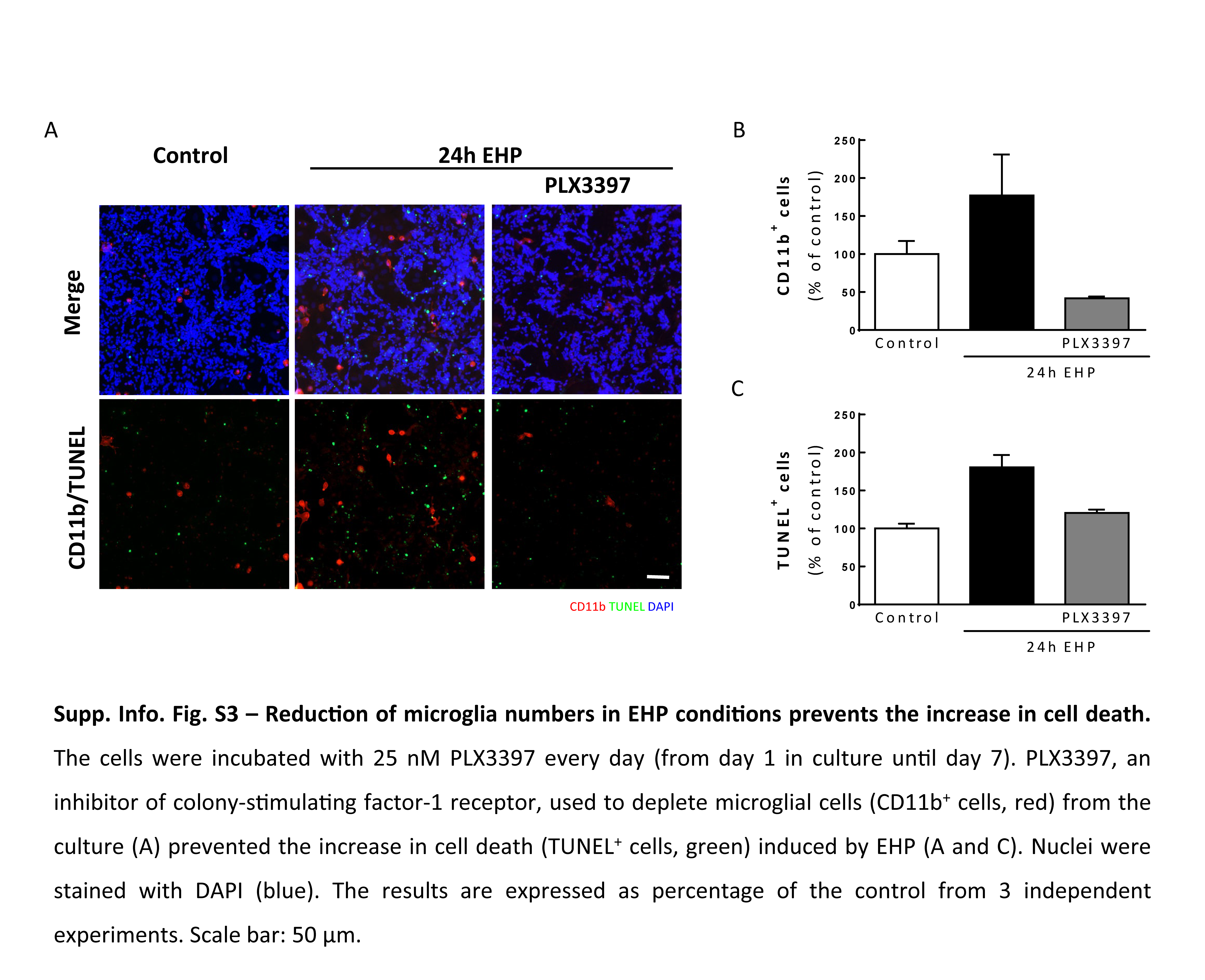

Supplement: Supplementary file 3 — Figure S3 Reduction of microglia numbers in EHP conditions prevents the increase in cell death. The cells were incubated with 25 nM PLX3397 every day (from day 1 in culture until day 7). PLX3397, an inhibitor of colony‐stimulating factor‐1 receptor, used to deplete microglial cells (CD11b* cells, red) from the culture (a) prevented the increase in cell death (TUNEL* cells, green) induced by EHP (a and c). Nuclei were stained with DAPI (blue). The results are expressed as percentage of the control from three independent experiments. Scale bar: 50 μm. Number of microglial cell (Cd11b‐immunoreactive cells) in culture after PLX3397 incubation. [file GLIA-67-896-s003.tif]

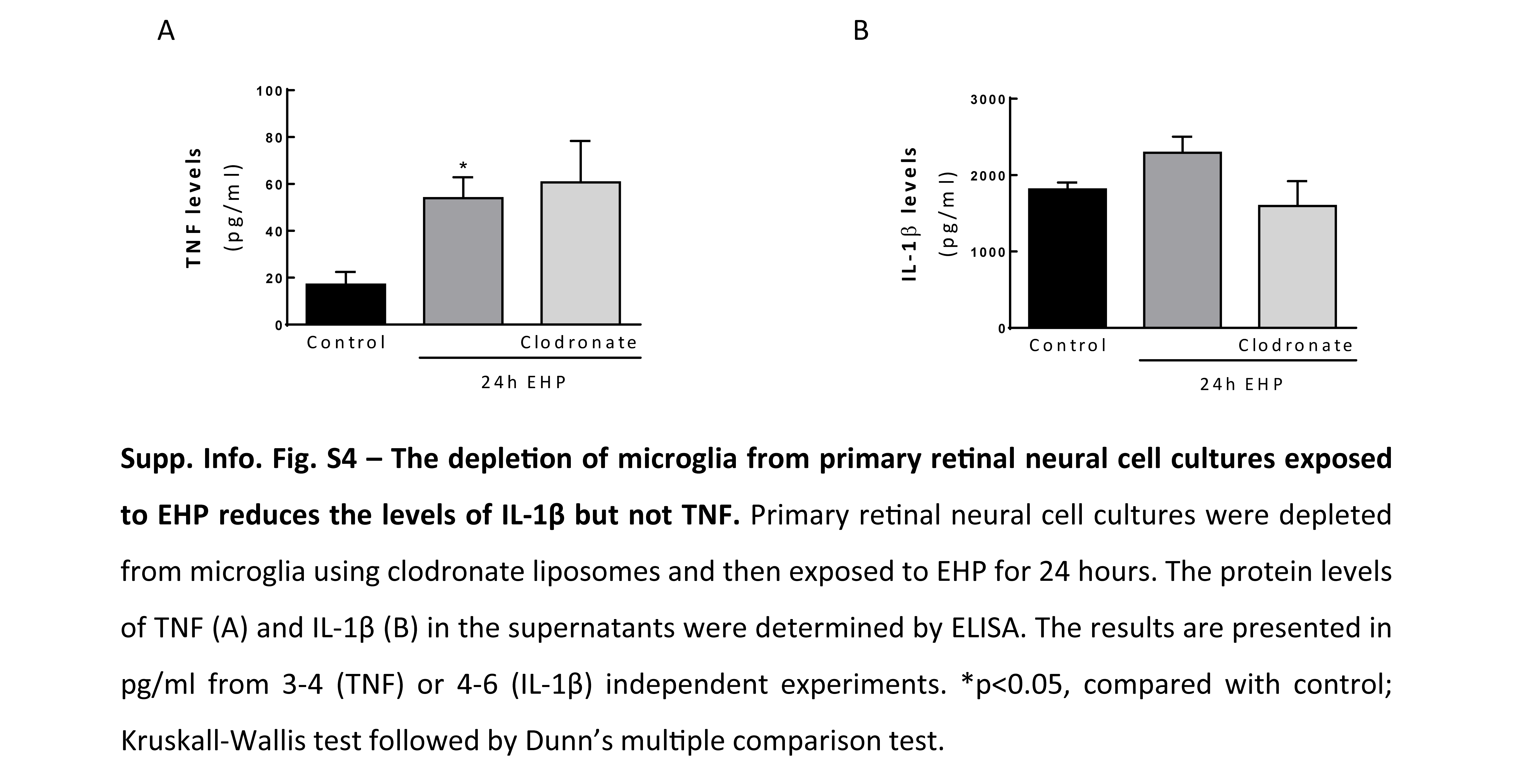

Supplement: Supplementary file 4 — Figure S4 The depletion of microglia from primary retinal neural cell cultures exposed to EHP reduces the levels of IL‐1B but not TNF. Primary retinal neural cell cultures were depleted from microglia using clodronate liposomes and then exposed to EHP for 24 hr. The protein levels of TNF (a) and IL‐1B (b) in the supernatants were determined by ELISA. The results are presented in pg/mL from 3 to 4 (TNF) or 4 to 6 (IL‐1B) independent experiments. *p < 0.05, compared with control; Kruskal–Wallis test followed by Dunn's multiple comparison test. [file GLIA-67-896-s004.tif]
